# Morphological profiling in human neural progenitor cells classifies hits in a pilot drug screen for Alzheimer’s disease

**DOI:** 10.1093/braincomms/fcae101

**Published:** 2024-03-28

**Authors:** Amina H McDiarmid, Katerina O Gospodinova, Richard J R Elliott, John C Dawson, Rebecca E Graham, Marie-Therese El-Daher, Susan M Anderson, Sophie C Glen, Simon Glerup, Neil O Carragher, Kathryn L Evans

**Affiliations:** Centre for Genomic & Experimental Medicine, Institute of Genetics & Cancer, University of Edinburgh, Western General Hospital, Edinburgh EH4 2XU, UK; Centre for Genomic & Experimental Medicine, Institute of Genetics & Cancer, University of Edinburgh, Western General Hospital, Edinburgh EH4 2XU, UK; Cancer Research UK Scotland Centre, Institute of Genetics & Cancer, University of Edinburgh, Western General Hospital, Edinburgh EH4 2XU, UK; Cancer Research UK Scotland Centre, Institute of Genetics & Cancer, University of Edinburgh, Western General Hospital, Edinburgh EH4 2XU, UK; Cancer Research UK Scotland Centre, Institute of Genetics & Cancer, University of Edinburgh, Western General Hospital, Edinburgh EH4 2XU, UK; Medical Research Council Human Genetics Unit, Institute of Genetics & Cancer, University of Edinburgh, Western General Hospital, Edinburgh EH4 2XU, UK; Centre for Genomic & Experimental Medicine, Institute of Genetics & Cancer, University of Edinburgh, Western General Hospital, Edinburgh EH4 2XU, UK; Centre for Genomic & Experimental Medicine, Institute of Genetics & Cancer, University of Edinburgh, Western General Hospital, Edinburgh EH4 2XU, UK; Department of Biomedicine, Aarhus University, 8000 Aarhus, Denmark; Cancer Research UK Scotland Centre, Institute of Genetics & Cancer, University of Edinburgh, Western General Hospital, Edinburgh EH4 2XU, UK; Centre for Genomic & Experimental Medicine, Institute of Genetics & Cancer, University of Edinburgh, Western General Hospital, Edinburgh EH4 2XU, UK

**Keywords:** *SORL1*, Alzheimer’s disease, induced-pluripotent stem cell, drug screen, morphological profiling

## Abstract

Alzheimer’s disease accounts for 60–70% of dementia cases. Current treatments are inadequate and there is a need to develop new approaches to drug discovery. Recently, in cancer, morphological profiling has been used in combination with high-throughput screening of small-molecule libraries in human cells *in vitro*. To test feasibility of this approach for Alzheimer’s disease, we developed a cell morphology-based drug screen centred on the risk gene, *SORL1* (which encodes the protein SORLA). Increased Alzheimer’s disease risk has been repeatedly linked to variants in *SORL1*, particularly those conferring loss or decreased expression of SORLA, and lower *SORL1* levels are observed in post-mortem brain samples from individuals with Alzheimer’s disease. Consistent with its role in the endolysosomal pathway, *SORL1* deletion is associated with enlarged endosomes in neural progenitor cells and neurons. We, therefore, hypothesized that multi-parametric, image-based cell phenotyping would identify features characteristic of *SORL1* deletion. An automated morphological profiling method (Cell Painting) was adapted to neural progenitor cells and used to determine the phenotypic response of *SORL1^−/−^* neural progenitor cells to treatment with compounds from a small internationally approved drug library (TargetMol, 330 compounds). We detected distinct phenotypic signatures for *SORL1^−/−^* neural progenitor cells compared to isogenic wild-type controls. Furthermore, we identified 16 compounds (representing 14 drugs) that reversed the mutant morphological signatures in neural progenitor cells derived from three *SORL1^−/−^* induced pluripotent stem cell sub-clones. Network pharmacology analysis revealed the 16 compounds belonged to five mechanistic groups: 20S proteasome, aldehyde dehydrogenase, topoisomerase I and II, and DNA synthesis inhibitors. Enrichment analysis identified DNA synthesis/damage/repair, proteases/proteasome and metabolism as key pathways/biological processes. Prediction of novel targets revealed enrichment in pathways associated with neural cell function and Alzheimer’s disease. Overall, this work suggests that (i) a quantitative phenotypic metric can distinguish induced pluripotent stem cell-derived *SORL1*^−/−^ neural progenitor cells from isogenic wild-type controls and (ii) phenotypic screening combined with multi-parametric high-content image analysis is a viable option for drug repurposing and discovery in this human neural cell model of Alzheimer’s disease.

## Teaser

Loss of *SORL1* was used to model Alzheimer’s disease. Cell Painting (a high-content imaging assay) was used to profile morphology in vehicle-treated wild-type and vehicle- and compound-treated SORLA-depleted neural progenitor cells. Screening 330 small molecules, McDiarmid *et al*. pilot machine learning-based hit classification for phenotypic drug discovery in Alzheimer’s disease.

## Graphical Abstract

**Graphical Abstract:**
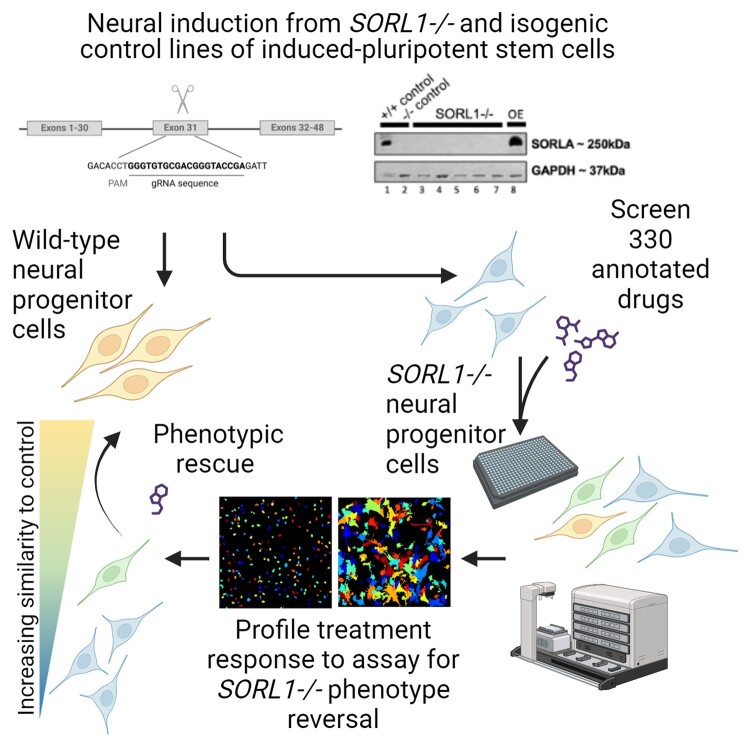

## Introduction

Alzheimer’s disease is the most common form of dementia. It is associated with a progressive decline in cognition, ultimately leading to incapacitation and death.^1^ Toxic amyloid-beta (Aβ) proteins, Aβ_40_ and Aβ_42_, produced by amyloidogenic processing of the amyloid precursor protein (APP) form insoluble, extracellular plaques in the brain of cases.^2^ Aβ-related pathology, intracellular neurofibrillary tangles of hyperphosphorylated tau protein^3^ and neuroinflammation^4^ are thought to lead to disruption of neuronal function and neurodegeneration in brain regions important for cognition (for example, the hippocampus). Currently, there is no cure, although modest, temporary relief from the cognitive impairment is achieved in a proportion of cases with acetylcholine esterase inhibitors (e.g. rivastigmine)^5,6^ or the *N*-methyl-D-aspartate receptors antagonist memantine.^7^ No new drugs have been licensed since memantine in 2002 and only two new potential treatments, immunotherapeutics aducanumab and lecanumab, which target Aβ aggregates in the brain, have been discovered in the last two decades.^8-11^ Whilst aducanumab reduced plasma levels of Aβ_40_ and Aβ_42_ in a dose-dependent manner,^8^ it was not associated with significant improvements to cognition or function in participants.^10^ Results from the lecanumab trial suggest treatment has modest beneficial effects on disease progression over 18 months, but adverse outcomes such as reaction to infusion and effusions or oedema^11^ were observed in substantial numbers of participants. Thus, there is the opportunity to explore alternative targets/pathways and treatments, via drug discovery and repositioning, rather than solely focussing on clearance of Aβ plaques.

Genetic, clinical and functional analyses strongly support involvement of the sortilin-related receptor 1 (*SORL1*, which encodes SORLA) in Alzheimer’s disease. In genome-wide association analyses, the associated variants in *SORL1* are largely non-coding and are expected to exert their effects via altering expression level.^12^ Haploinsufficiency of *SORL1* is highly penetrant in Alzheimer’s disease, with 2% of early-onset cases being attributed to rare *SORL1* loss-of-function variants.^13,14^ Loss-of-function mutations in *SORL1* are associated with >50% Alzheimer’s disease penetrance for individuals carrying two copies of *APOE* ε3 at >75 years of age, and 100% for those homozygous for APOE ε4.^15^  *SORL1* risk variants have also been shown to predict endophenotypes (decreased white matter fractional anisotropy and increased amyloid pathology in post-mortem brain) in dementia-free individuals.^16^ Decreased expression of SORLA is found in the brains of cases *post-mortem* and evidence suggests this occurs prior to the development of clinical disease.^17,18^ It can be concluded, therefore, that reduced SORLA expression increases risk in late/early-onset sporadic and familial cases Alzheimer’s disease.

SORLA is a member of the VPS10p-domain receptor gene family of multi-functional neuronal proteins (other members: Sortilin and SORCS1–3). It shuttles cargo, including a number of molecules that are important to Alzheimer’s disease pathology^19-22^ between the plasma membrane, endosomes, lysosomes and the trans-Golgi network. The trafficked molecules include APP, which is shuttled from endosomes to the Golgi, decreasing the production of Aβ. SORLA also sorts Aβ peptides to lysosomes, where they are degraded. Decreased *Sorl1* expression in mice accelerates Aβ production and plaque deposition,^17,23^ while overexpression of a human *SORL1* cDNA significantly reduced the amount of murine Aβ in wild-type mice, and human Aβ in an APP-transgenic mouse model of Alzheimer’s disease.^24^  *Sorl1* haploinsufficiency in mini-pigs induced a cerebrospinal fluid biomarker profile identical to that seen in Alzheimer’s disease.^25^

Endolysosomal pathways are important in the pathogenesis of neurodegenerative conditions.^21,26-28^ Loss of SORLA leads to morphological and functional abnormalities in organelles from this pathway.^19,20^ In a minipig model, *S*orl*1* haploinsufficiency induced endosomal enlargement in neurons.^25^ Knupp *et al*.,^20^ showed that depletion of *SORL1* led to enlargement of early endosomes (independent of amyloidogenic APP processing) in SORLA-depleted human iPSC-derived neural progenitor cells (NPCs) and neurons, but not microglia. Similarly, Hung *et al*.,^19^ found that loss of *SORL1* in iPSC-derived neurons resulted in endosome, lysosome and autophagy defects. These findings suggest that morphological phenotypes quantified using fluorescence microscopy distinguish between SORLA-depleted and wild-type NPCs and neurons.

NPCs are a relevant cell type for research into Alzheimer’s disease. A developmental basis for Alzheimer’s disease has been hypothesized. Some proteins implicated in neurodegeneration/Aβ-pathology have important developmental functions.^29,30^ Atypical neurodevelopmental trajectories have been described in carriers of *SORL1* risk variants,^16^ and there is evidence for increased proliferation of NPCs in mice lacking Sorla.^31^ In addition, to being key to neurodevelopment, recent studies have shown that NPCs and hippocampal neurogenesis persist beyond 90 years of age in Alzheimer’s disease cases.^32,33^ Hippocampal neurogenesis declined with age, with the extent of decline being correlated with disease severity. The persistence of NPCs into old age, and the correlation between decline and disease, suggest an important role for NPCs in Alzheimer’s disease. In addition, NPCs are an ideal starting point for developing high-throughput drug screening, not only because prior reports outline relevant phenotypes that can be observed using image analysis but also because derivation of NPCs in large quantities is time- and cost-effective.

Given the above considerations, we developed a phenotypic drug screen using *SORL1*^−/−^ NPCs. Our screen was designed to identify compounds that caused reversion of the *SORL1* knock-out phenotype to that of the healthy (isogenic) control NPC line. We used the morphological profiling assay, Cell Painting, a fluorescent image-based profiling approach that permits hypothesis-free and relatively unbiased interrogation of phenotypic features. CellProfiler was used to quantify >1000 cellular and sub-cellular morphological features from this data.^34^ Dimensionality reduction, multivariate statistical analysis (including distance metrics and classification by machine learning) was then used to quantify phenotypic differences between mutant and healthy control cells. Subsequently, machine learning analysis methods were applied to identify compounds that induce reversion of the quantitative phenotypic signature of mutant cells towards that of wild-type healthy cells. Such image-based multi-parametric, computational approach may overcome some of the limitations of traditional single-readout end-point drug discovery assays.^35^

Here, we show that morphological profiling by Cell Painting robustly classifies wild-type NPCs from those lacking SORLA. In addition, we have performed a pilot drug screen with a small library comprising FDA/internationally approved, biologically annotated small molecules. The screen yielded hits that reversed the mutant phenotype, demonstrating the potential of this assay to profile treatment response and identify compounds relevant to SORLA-related pathology and potentially Alzheimer’s disease.

## Materials and methods

### Human-induced pluripotent stem cell line

Human-induced pluripotent stem cell (hiPSC) line WTSIi004-B (QOLG-1) was obtained from the European Bank for Induced Pluripotent Stem Cells (ebisc.org) under a material transfer agreement and access use agreement. WTSIi004-B line was derived from fibroblasts from a healthy male donor aged 35–39 that met the following criteria: normal karyotype confirmed in hiPSCs, homozygous for *APOE* e3/e3 (which allows effect of *SORL*1 risk to be explored without confounding factors associated with risk *APOE* ε4 genotypes) and confirmed differentiation into neural cells.

### Generation of isogenic homozygous SORLA-depleted iPSCs by CRISPR-Cas9/genome editing

CRISPR-Cas9 guide RNAs (gRNAs) targeting exon 31 of the human *SORL1* gene (*SORL1*ex31) were designed using two open-source, computational tools; the Zhang Lab CRISPR Design website (https://crispr.mit.edu) and CHOPCHOP (https://chopchop.cbu.uib.no/). The final sequence was selected based on high specificity to the target site and low predicted off-target activity. The oligos were phosphorylated and cloned into px458, which expresses Cas9 endonuclease and GFP (RRID: Addgene_48138). Constructs were delivered using nucleofection. Fluorescence automated sorting was used to select GFP + cells which were seeded as single cells before sub-cloning and genotyping.

### *SORL1*ex31 genotyping assay, sequencing and assessment of sortilin expression

QuickExtract™ DNA Extraction Solution (QE09050, Lucigen) was used to extract gDNA from selected sub-clones for polymerase chain reaction (PCR). The gDNA samples were amplified by PCR using ReadyMix(TM) Taq PCR Reaction Mix (P4600, Sigma) containing 12.5 µl Reaction Mix (P4600, Merck, 12.5 µl), 2 µl 20 µM Primers 8.5 µl molecular grade and 2 µl Quick Extract gDNA sample. Five micro-litres ofPCR products were visualized on 1.5% agarose gels (1 × TBE Buffer) with ×10 loading dye alongside 10 µl 1 kb plus DNA Ladder. Products of the expected size were subject to sequence analysis. Mutation of the *SORL1*ex31 locus was confirmed by sequencing of the target locus in *SORL1* exon 31. Off-target cleavage sites were predicted computationally and sequenced. Karytypic normality was confirmed by KaryoStat Assay (ThermoFisher). Clones with predicted loss-of-function mutations were tested by immunoblotting with primary antibodies against SORLA (1:4000; 611680, BD Transduction Labs) and GAPDH (1:10 000; MAB374, Merck).

### Derivation of NPCs

Colonies of hiPSCs from *SORL1*ex31 sub-clones and parental wild-type clones were cultured until 70–80% confluent with no visible differentiation before dissociation and retrieval from suspension by centrifugation at 200 g for 3 min. Neural induction was achieved using the StemDiff Neural Induction Kit (IM, 08581, Stem Cell Technologies) and following the embryoid-body protocol as per manufacturer’s instructions. The derivation was performed in technical duplicate for 3 *SORL1*^−/−^ sub-clones and 3 isogenic wild-type controls from one biological iPSC donor line. NPC cultures were confirmed routinely tested for mycoplasma.

### Immunocytochemistry

NPCs were fixed using 4% PFA for 20 min after 48 h in culture in an optical 384-well plate. NPCs were washed twice with ×1 phosphate buffered saline (PBS) for 5 min per wash. Permeabilization and blocking was performed prior to incubation with antibodies (1 h in freshly prepared blocking solution [PBS with 5% (v/v) normal donkey serum (D9663, Sigma) and 0.3% (v/v) Triton X-100)]. Primary antibodies Anti-Sox2 (1:500 AB5603, Chemicon, stem cell marker), Anti-Nestin (1:500, MAB5326, Chemicon, NPC marker) and Anti-EEA1 (1:250, BD BioScience, 610456, endosome marker) were diluted in blocking solution. The primary antibodies were removed after overnight incubation at 4°C. NPCs were washed twice with ×1 PBS for 5 min per wash. Then, fluorescent-tagged secondary antibodies Alexa Fluor-488 Donkey Anti-Rabbit (Invitrogen) and Alexa Fluor-594 Donkey Anti-Mouse (Invitrogen) were diluted to 1:500 in blocking solution with DAPI (1 mg/ml). After incubation with secondary antibodies for 2 h at room temperature, NPCs were washed twice and stored in ×1 PBS (50 ml per well). Expression of these markers was validated in each NPC derivation in all *SORL1*^−/−^ sub-clones and wild-type in passage-matched cultures, isogenic controls with a total of three well-level and four image-level technical replicates.

### Drug screening

Three *SORL1*^−/−^ sub-clones and one parental WT (*n* = 1) NPCs at p6 were seeded (5000 cells per well) in 384-well optical-bottom microplates coated with Matrigel (Corning, 254234) in NPM. Nine plates were seeded, 3 per *SORL1*ex31 knock-out sub-clone. WT NPCs were seeded in 24 wells per plate as reference samples for phenotypic rescue and as within-plate 2% DMSO vehicle-treated controls. Plates were incubated for 24 h at 37°C, 95% humidity and 5% CO_2_ before addition of compound treatments. The TargetMol Annotated Anti-Cancer library (L2110, 330 compounds) was supplied in assay-ready plates and thawed fresh on the morning of use. Serial dilution of the stock library was achieved using Biomek FX liquid handling system to generate compound plates at working concentrations suitable for in-well dilution in the NPC screening plates at three concentrations: 100, 300 nM and 1 µM. Thus, each sub-clone was screened across three concentrations in three plates (totalling 9 plates). A total of 330 compounds were screened using one compound treatment per well per plate with 4 image-level replicates per well. Compound treatments were tested at three concentrations (100, 300 nM and 1 mM) in three *SORL1*^−/−^ NPC sub-clonal lines with in-plate vehicle-treated *SORL1*^−/−^ and wild-type, vehicle-treated controls.

### Cell Painting assay

NPCs were fixed in 4% PFA for 20 min at room temperature. NPCs were washed twice with ×1 PBS for 5 min per wash. Fluorescent dyes were diluted in 1% (v/v) BSA solution in ×1 PBS with 0.1% (v/v) Triton X-100 to prepare the Cell Painting staining solution (Table 1). The Cell Painting Staining solution was applied to each well of the nine 384-well optical-bottom microplates (20 µl per well) and incubated for 1 h at room temperature protected from light to preserve fluorescence. Cells were washed twice with ×1 PBS and then ×1 PBS added to each well (50 µl per well) and a foil plate seal applied prior to imaging using florescence detection microscopy.

**Table 1:** Cell Painting assay reagents with conjugated fluorophore emission/excitation spectra filters, cellular components label and ImageXpress**™** XL channel used for image acquisition corresponding to each dye

Dye ​	Filter (excitation; nm) ​	Filter (emission; nm) ​	Organelle or cellular component ​	Imaging channel ​	Dilution/concentration	Manufacturer​	Cat. no.​
DAPI ​	387/11 ​	417–477 ​	Nucleus ​	DAPI ​	1μg/mL ​	ThermoFisher​	62248 ​
Concanavalin A/Alexa Fluor 488 conjugate​	472/30 ​	503–538 ​	Endoplasmic reticulum ​	FITC ​	20 µg/mL​	Invitrogen​	C11252​
SYTO 14 green fluorescent nucleic acid stain ​	531/40 ​	573–613 ​	Nucleoli, cytoplasmic RNA ​	Cy3 ​	3μM ​​	Invitrogen​	S7576​
Phalloidin/Alexa Fluor 568 conjugate, wheat- germ agglutinin/Alexa Fluor 555 conjugate ​	562/40 ​	622–662 ​	F-actin cytoskeleton, Golgi apparatus, plasma membrane ​	TxRed ​	1:500 (Phalloidin)​​	Abcam (Phalloidin)​​	ab176757 (Phalloidin)​​
					2μg/mL ​ (WGA)​	Invitrogen (WGA)​	W11262 ​(WGA)​
MitoTracker Deep Red ​	628/40 ​	672–712 ​	Mitochondria ​	Cy5 ​	600 nM ​	Invitrogen​	M22626 ​

DAPI, 4′,6-diamidino-2-phenylindole; ng, nanograms; nm, nanometres; TxRed, Texas Red; WGA, wheat-germ agglutinin.

### Automated fluorescence microscopy

Fluorescence imaging was performed using ImageXpress Confocal (Molecular Devices, USA) and accompanying MetaXpress Software (Molecular Devices, USA) with a robotic plate handling arm (Harmony, peak analysis and automation, UK). User-defined parameters (such as exposure time and laser off-set) were optimized based on a sample of 10 wells randomly distributed across each of the 384-well microplates (781091, Greiner) and kept constant between plates thereafter. For immunocytochemistry, images were acquired for three fluorescent channels (filters: DAPI, FITC and Texas Red) at ×20 magnification. For Cell Painting, images were acquired for five fluorescent channels (filters: DAPI, FITC, Cy3, Texas Red and Cy5, Table 1) at ×20 magnification. For each well, four fields of view were captured (image-level quadruplicate). Illumination correction was achieved using a within-instrument tool to adjust for small variations in sample illumination for each field of view. Illumination correction was applied at time of image acquisition to correct intensity values according to an illumination correction function calculated using MetaXpress Software.

### Image analysis

Quantitative image analysis of fluorescence microscopy images (from both immunocytochemistry samples and Cell Painting assay) was performed using CellProfiler (cellprofiler.org, v4.1.2). Briefly, the region of interest, i.e. a cell, was defined by segmenting nuclei from each of the four image-level replicates acquired based on DAPI + objects (from DAPI-channel images) to generate a nuclear mask. Using the nuclear mask as a reference point for each cellular object, segmentation of the cell body was performed to define the cellular region of interest. Those cellular regions of interest were then used to isolate cellular objects within each image that were subjected to either classification as positive or negative for a specific marker (Sox2/Nestin) or in the case of Cell Painting to multi-parametric analysis to quantify morphological features per object per image using a bespoke pipeline (CellProfiler, v4.2.1) which included, but was not limited to, measures of stain colocalization, object adjacency, size, shape, area, texture, radial distribution, granularity and intensity.

### Data analysis and statistics

CellProfiler analysis was submitted as an array job using scripts data staging analysis and data destaging GridEngine scripts generated by cptools2 (https://github.com/CarragherLab/cptools2), on ‘Eddie’ the high-performance computing cluster at University of Edinburgh. The quantitative multivariate datasets were exported in.csv format, and analysed using HC StratoMineR (Core Life Analytics). The resulting data set comprised of morphological profiles from three sub-clonal well-level replicates for each compound treatment at each concentration (uniplicate) with four image-level replicates per well. Raw data is publicly available (https://idr.openmicroscopy.org/, accession to be confirmed).

Principal component analysis (PCA) was used for dimensionality reduction and factor analysis was applied to extract components important for explaining variation with reference to the samples and controls. Variables causing singularity were eliminated. Factor analysis was applied with respect to with the DMSO vehicle-treated *SORL1*^−/−^ and DMSO vehicle-treated WT NPCs. This was performed using oblique (oblimin) factor rotation method and factor scores calculated by 10 Berge method. Using Kaiser’s criterion and examination of a Scree plot, a set of 50 principal components (PCs) were selected for analysis. Euclidean distance and Bray–Curtis dissimilarity metrics were used to test for phenotypic separation of *SORL1*^−/−^ and wild-type controls on the 50 PCs. Selected PCs were also used as quantitative signatures in a machine learning classification model for hit selection. Random Forest, neural network (NN) and support vector machine (SVM) classifier algorithms were trained on the vehicle (DMSO) treated positive and negative control images (20% test versus 80% training set) and each model was used to classify sample (compound treatment) images as either *SORL1*ex31 knock-out or wild-type. A three-layer NN with size and decay as hyper-parameters resulted in greatest separation of morphological signatures and was therefore selected as the method for hit selection. All algorithms were applied to detect objects with morphological profiles similar to the positive control. Briefly, the positive control used phenotypic profiles from the DMSO vehicle-treated wild-type NPCs and was used as the focus class in the classifier to determine which compound-treated *SORL1*ex31 knock-out NPCs classified as more similar (>50.5% likelihood) to the wild-type positive control following treatment. Graphical visualizations were produced using Plotly in R (v4.0.2, www.r-project.org).

### Transcriptomic profiling

Transcriptome expression was profiled using RNAseq in three, sub-cultured passages of wild-type NPCs (p2, p4 and p5) from the parental WT hiPSC line (QOLG-1). The dataset was profile was used to filter gene nodes not expressed in our cell lines from the network analysis, and for background correction during enrichment analysis.

### Network analysis

Validated and predicted target lists for each compound hit were generated using database searches. Experimentally validated compound–protein interactions were generated by searching the compound names (grouped by mechanism of action according to published functional annotations) in the STITCH database (STITCH: http://stitch.embl.de/). A predicted target list for each compound hit was generated using a similarity ensemble approach (SEA) search (sea.bkslab.org).^36^ The SEA search uses the SMILES string for each compound to query possible compound–protein interactions not limited to experimental annotations. By exploring the pharmacological space corresponding to a particular chemical structure it is possible to computationally predict possibly novel protein targets. Those putative interactions predicted by SEA were ranked according to significance, which is a measure of the probability of the predicted interaction, as well as Tanimoto coefficient (MaxTC), a measure of structure-based similarity.

### Enrichment analysis

Enrichment analysis was conducted using the enrichment tool included in the STRING platform to determine which predicted and validated network components (i.e. proteins/genes) were over-represented in biological pathways (KEGG database, https://www.genome.jp/kegg/), biological processes and molecular functions (Gene Ontology; http://geneontology.org/).^37^ The STRING database was used to highlight nodes representing genes associated with KEGG/GO terms within networks.

## Results

### Exploring mechanisms associated with SORLA depletion in NPCs

**Figure 1:**
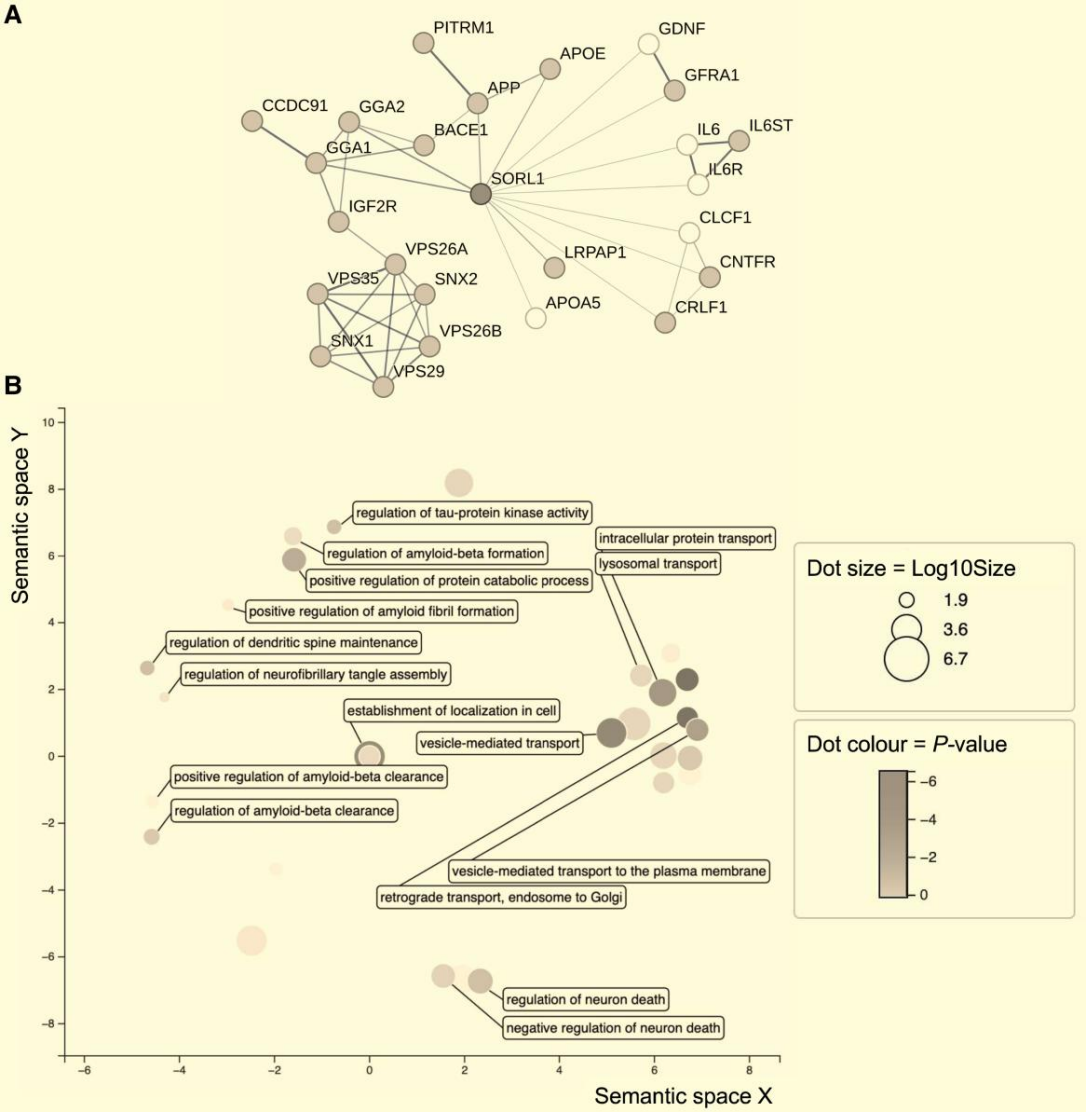
**The molecular mechanisms associated with SORLA depletion are enriched in Alzheimer’s disease relevant biological processes**. (**A**) Multiple proteins known to interact with SORLA based on experimental evidence from the STRING database were expressed at transcriptomic-level in our NPCs (expressed genes shown in dark grey). Network diagram lines indicate physical interaction based on data from experimental evidence and databases. Line weight/thickness indicates confidence of interaction. (**B**) Enrichment of the network of NPC-expressed SORLA interactors (dark grey nodes from the network diagram in **A** revealed significant overlap between genes interacting with SORLA, and those in Gene Ontology Biological Processes associated with endolysosomal sorting and amyloid processing (visualization by semantic similarity generated by ReviGo). Dot size represents log_10_size where size is equal to the number of annotations in the Gene Ontology Term. Dot colour represents the scaled *P-*value (*P =* 0.05 set to 0) from the enrichment test for SORLA interaction network gene set with given Gene Ontology Term. NPC, neural progenitor cell.

The STRING database was used to generate a network of NPC-expressed proteins that interact with SORLA (expression was determined by RNA-sequencing of wild-type NPCs, grey nodes in Fig. 1A indicate genes expressed in the QOLG-1 donor line). We tested for enrichment of biological pathways amongst the expressed genes (Supplementary Table 1). Significant enrichment was observed in multiple AD- and SORLA-related Gene Ontology Biological Processes (GOBP) terms (Fig. 1B), e.g. vesicle-mediated transport to the plasma membrane [GOBP:0016192, Hypergeometric test, effect size (ES) = 0.87, Benjamini–Hochberg false discovery rate (FDR) correction, *P* = 1.9 × 10^−5^], Golgi vesicle transport (GOBP:0048193, Hypergeometric test, ES = 1.24, FDR, *P* = 0.00047), regulation of Aβ formation (GOBP:1902003, Hypergeometric test, ES = 1.92, FDR, *P* = 4.2 × 10^−3^) and neurofibrillary tangle assembly (GOBP:1902996, Hypergeometric test, ES = 2.84, FDR, *P* = 3.0 × 10^−3^).

### Generating SORLA-depleted hiPSCs for derivation of NPCs

**Figure 2:**
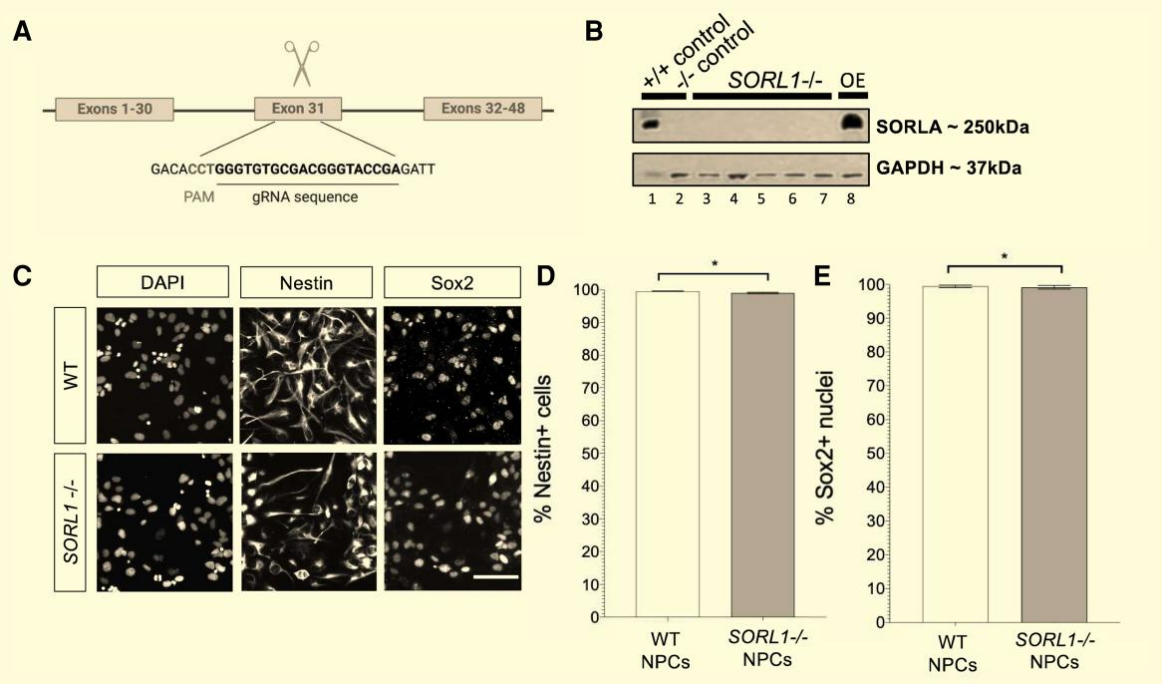
**Depletion of SORLA by targeting of exon 31 in *SORL1***. (**A**) A single-guide RNA was used to target CRISPR-Cas9 cleavage at a site within exon 31 of the *SORL1* gene to induce a homozygous mutation by non-homologous end-joining (schematic produced using BioRender). (**B**) Mutations introduced using this method resulted in depletion of SORLA expression beyond levels detectable by immunoblotting by SORLA primary antibody with GAPDH loading control (Lane 1 = unedited wild-type positive control, Lane 2 = negative control, Lanes 3–7 = multiple SORLA-depleted sub-clones with Lanes 4–6 representing *SORL1*^−/−^ sub-clones utilized in the current study and Lane 8 = SORLA overexpression in HEK cells as a positive control). (**C**) Representative grey-scale images of NPCs from wild-type and *SORL1*^−*/−*^ with immunocytochemistry used to detect Sox2 (stem cell marker) and Nestin (neural progenitor cell marker). (**D**) Quantification showed no change in the relative proportion of Nestin + showed when comparing wild-type with *SORL1*^−*/−*^ NPCs (Mann–Whitney U test) based on 4 image-level replicates per well with 3–4 well-level replicates per sub-clone. (**E**) A significant decrease in the proportion of Sox2 + cells was found in *SORL1*^−*/−*^ NPCs compared to wild-type (Mann–Whitney U, *P* < 0.0001). Scale bar represents 50 µm. Non-parametric testing was applied due to unequal variance and non-Gaussian distribution of cell proportion quantification. Graphed data was grouped according to genotype. The bar representing WT isogenic NPCs shows quantification of the median cell marker proportions from a pool of 88 images acquired from 2 wild-type sub-clones from the CRISPR-Cas9 editing to mutate SORL1 exon 31 and 1 parent wild-type line. The bar representing SORL1^−/−^ NPCs shows median cell marker proportions from a pool of 88 images acquired from the 3 SORL1^−/−^ sub-clones from the CRISPR-Cas9 editing to mutate SORL1 exon 31. Error bars show range. * *P* < 0.05. WT, wild-type; DMSO, dimethyl sulfoxide; NPC, neural progenitor cell; DAPI, 4′,6-diamidino-2-phenylindole.

An hiPSC line from a healthy donor was selected for gene-editing as we wished to assay for phenotypic reversion from a genetic perturbation which mimics disease risk. Targeting exon 31 of *SORL1* in a human iPSC line (Fig. 2A) generated multiple SORLA-depleted sub-clones (Fig. 2B; Supplementary Fig. 1A). SORLA-depleted NPCs were derived from three sub-clones, two wild-type sub-clones isolated from the pool of gene-edited cells (crWT) and a parental wild-type (WT) (Fig. 2C). No genome-wide karyotypic abnormalities were observed, nor were mutations found at the top nine predicted off-target sites. WT and *SORL1*^−/−^ NPC cultures had >99% Nestin+/Sox2 + cells demonstrating efficient neural induction from iPSC. A decrease in the proportion of Nestin + cells (WT median proportion Nestin + cells = 99.77%; *SORL1*^−/−^ median proportion Nestin + cells = 99.58%, Mann–Whitney U = 3061*, P* = 0.0135, Fig. 2C and D; Supplementary Table 2, Supplementary Fig. 1B) and Sox2 + nuclei was observed in *SORL1*^−/−^ NPCs (WT median proportion Sox2 + nuclei 100.00 = %; *SORL*1^−/−^ median proportion Sox2 + nuclei = 99.82%, Mann–Whitney U = 1432*, P* = 0.0310, Fig. 2C and E; Supplementary Table 2, Supplementary Fig. 1B) suggesting SORLA depletion induces differences in pluripotency. To validate the increased endosome area and intensity previously observed in SORLA-depleted NPCs,^20^ early endosomes were detected and area/intensity quantified using fluorescence microscopy (Supplementary Fig. 2).

### Cell Painting assay labels cellular components in wild-type and *SORL1*^−/−^ NPCs in a pilot drug screen

**Figure 3:**
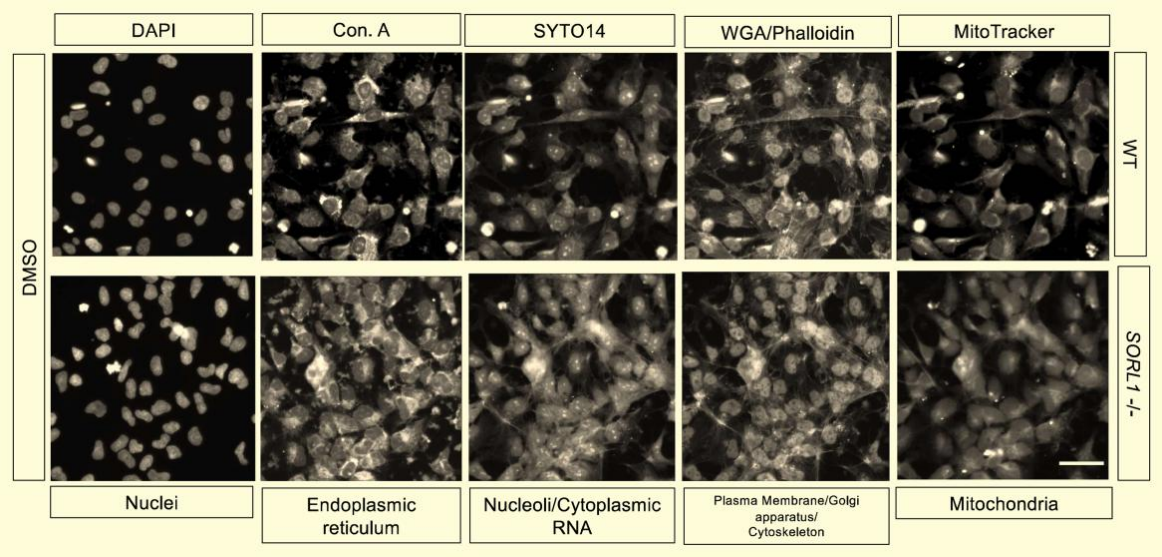
**Cell Painting using conjugated cell compartment-specific dyes in wild-type and *SORL1*^−*/−*^ NPCs**. Monolayer, adherent NPCs were fixed (4% PFA) and a multiplexed reaction mix containing fluorescent conjugated dyes specific to various cellular and sub-cellular compartments applied to cells with subsequent image capture using the high-throughput, automated, confocal fluorescence microscopy platform ImageXpress Micro Confocal (×20). These grey-scale images demonstrate fluorescent labelling of nuclei by DAPI, endoplasmic reticulum by Concanavalin A, nucleoli/cytoplasmic RNA by SYTO14, plasma membrane and Golgi apparatus by wheat-germ agglutinin, cytoskeleton by Phalloidin and mitochondria by MitoTracker in DMSO wild-type and SORL1^−/−^ NPCs and as such represent vehicle-treated positive and negative controls in the drug screening assay. Four sites per well were acquired providing 4 image-level replicates with 24 and 16 well-level replicates for the DMSO-treated WT and SORL1^−/−/^ NPCs, respectively. Scale bar represents 50 µm. WT, wild-type; DMSO, dimethyl sulfoxide; RNA, ribonucleic acid; Con. A, Concanavalin A; WGA, wheat-germ agglutinin; NPC, neural progenitor cell; DAPI, 4′,6-diamidino-2-phenylindole; PFA, paraformaldehyde.

To screen for compounds that rescue morphological profiles, *SORL1*^−/−^ NPCs were treated with one of 330 compounds selected from the TargetMol Annotated Compound Set (L2110, targetmol.com; Supplementary Table 3) at three concentrations (100, 300 or 1000 nM) alongside vehicle-treated wild-type and *SORL1*^−/−^ NPCs. Following Cell Painting (Fig. 3, Table 1) and pre-processing to remove redundant variables, 756 quantitative measurements of cellular features were selected for further analysis (Supplementary Table 4). Of these, 408 variables were significantly different between groups (one-way *ANOVA* with Benjamini–Hochberg FDR correction, *P* < 0.05, Supplementary Fig. 3).

### *SORL1* ^−/−^ NPCs have distinct quantitative morphological profiles from wild-type control NPCs

**Figure 4:**
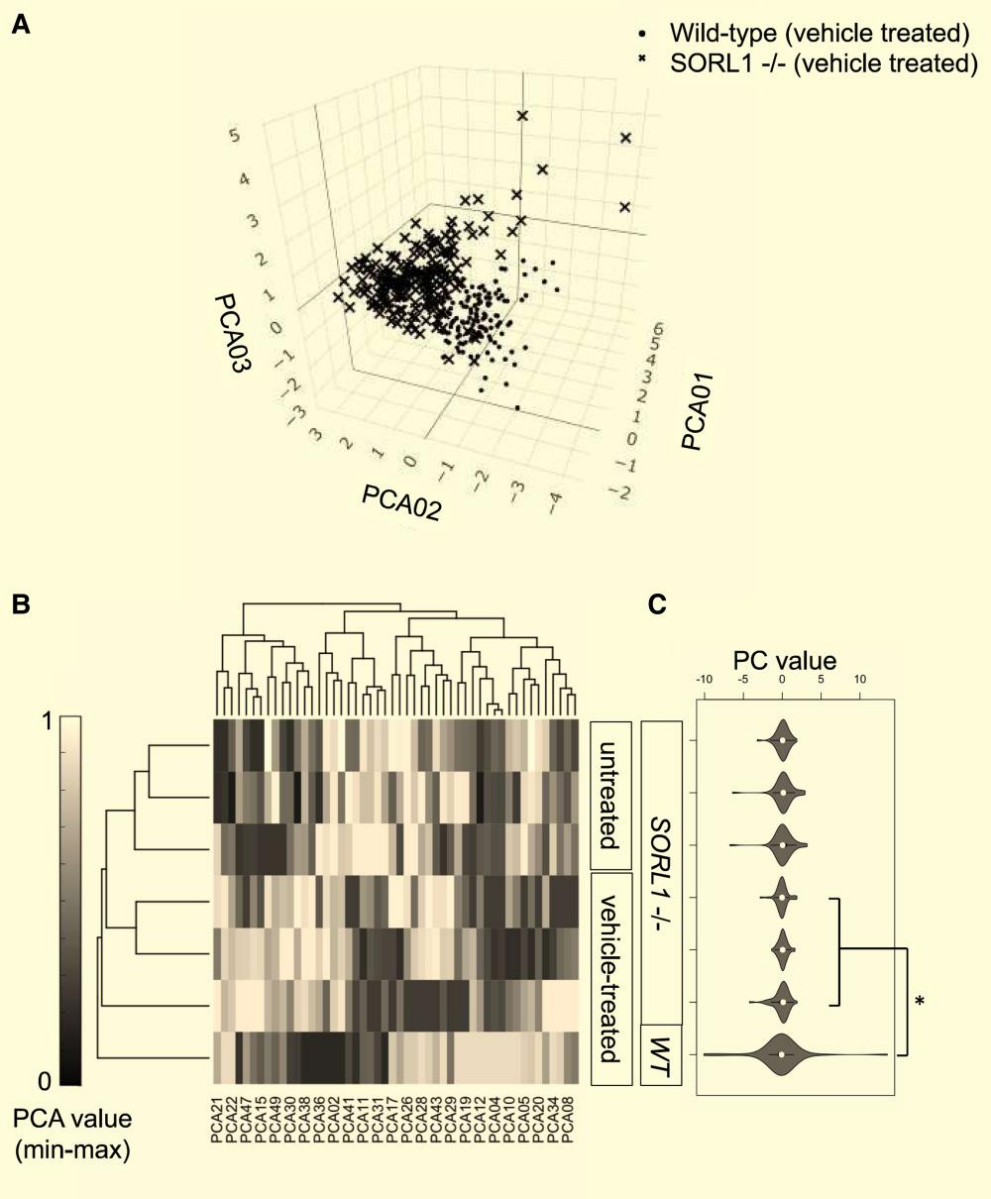
**Morphology of *SORL1*^−/−^ NPCs is distinct from isogenic wild-type controls**. (**A**) PCA was used to visualize phenotypic separation of vehicle-treated *SORL1*^−*/−*^  *NPCs* and isogenic wild-type controls in 3D. Each data point in the 3D scatter plot represents the well-level median (aggregated from 4 image-level replicates per well) for 24 and 16 well-level replicates acquired from WT and *SORL1*^−*/−*^ NPCs, respectively. PCA was then used to reduce dimensionality from 756 quantitative measures of cellular features from the Cell Painting assay to 50 phenotypic PCs. (**B**) Hierarchical clustering and heatmap visualization of 50 PCs (after min–max scaling) showing separation of the untreated and vehicle-treated NPCs *SORL1*^−*/−*^ NPCs, as well as separation of vehicle-treated *SORL1*^−/−^ NPCs from vehicle-treated wild-type NPCs. (**C**) Violin plots depict phenotypic signature of untreated and vehicle-treated NPCs *SORL1*^−*/−*^ NPCs and vehicle-treated wild-type NPCs based on unscaled PC values for PCs 1–50, and significant separation was observed in the screening control classes vehicle-treated, i.e. morphological signature of DMSO-treated *SORL1*^−*/−*^ NPCs was significantly different to that of the DMSO-treated wild-type NPCs. 3D, three-dimensions; DMSO, dimethyl sulfoxide; NPC, neural progenitor cell; PC, principal component; PCA, principal component analysis; WT, wild-type.

PCA was applied to reduce dimensionality of the 756 features to 50 non-redundant representative PCs (Supplementary Table 5). Fig. 4A is a visualization based on three-dimensional PCA. Hierarchical clustering of phenotypic signatures showed that untreated and vehicle-treated *SORL1*^−/−^ NPCs had similar morphology, which was distinct from that observed in the vehicle-treated wild-type control (Fig. 4B). Since Euclidean distance metrics did not perform well on this dataset, a non-Euclidean Bray–Curtis dissimilarity metric was applied to the morphological signatures for vehicle-treated wild-type, compound-treated, vehicle-treated and untreated *SORL1*^−/−^ NPCs based on the 50 PCs and demonstrated a significantly distinct phenotype between in control classes (Fig. 4C; Supplementary Fig. 4A, Bray–Curtis distance score = 1.27, *P =* 1.21 × 10^−2^).

### Classification predicts 16 compounds that reverse *SORL1*^−/−^ NPC phenotypic profiles towards that of wild-type NPCs

**Figure 5:**
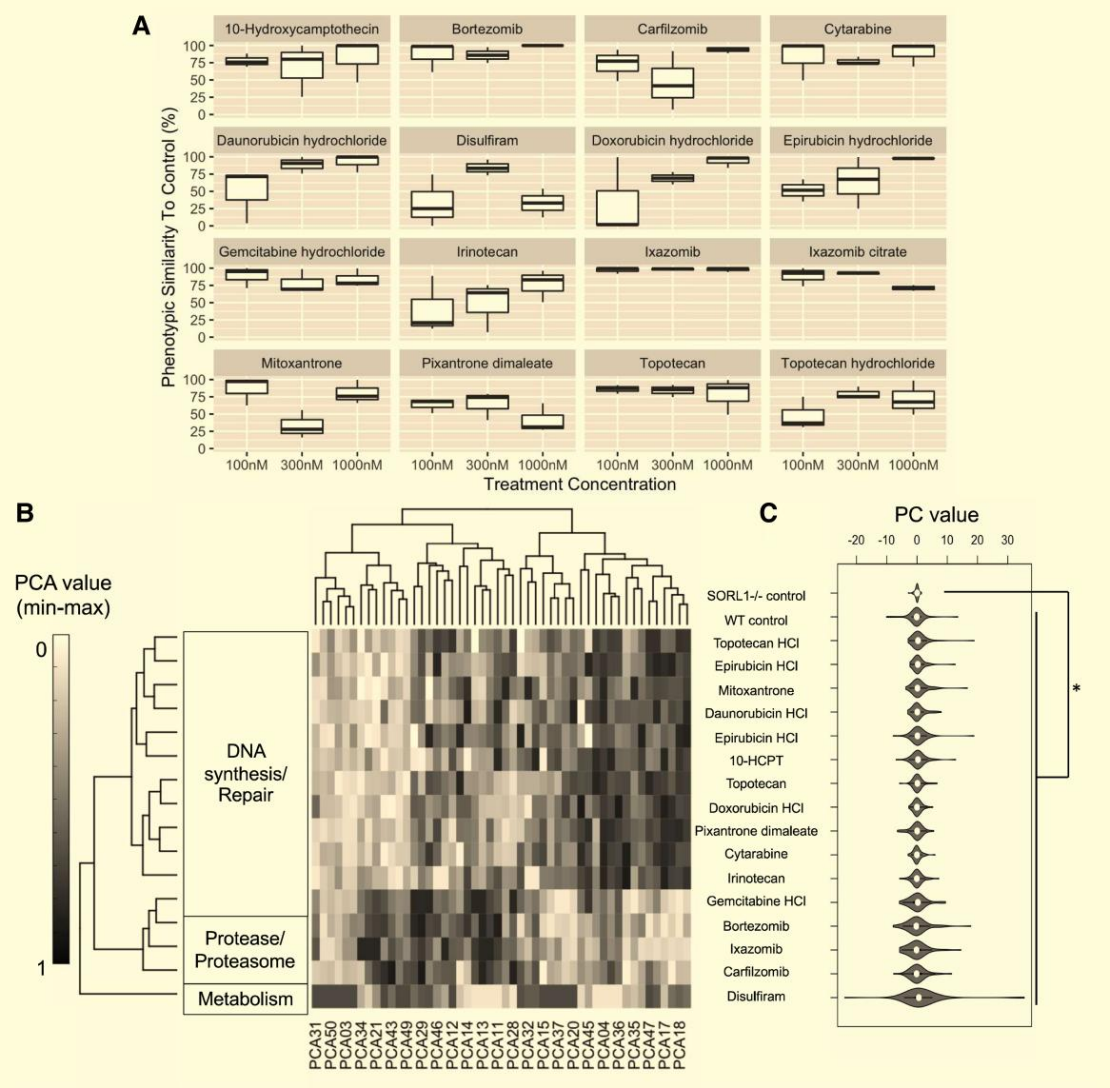
**Neural network classification predicts 16 hit compounds that induce significant changes to *SORL1*^−*/−*^ NPC morphology which increases similarity to isogenic wild-type controls**. Sixteen inhibitor compounds regulating three biological pathways/targets from five mechanistic classes from the 330-compound library were identified from the pilot drug screen. (**A**) Percentage likelihood of classification as a WT was used as a measure of phenotypic similarity to control (%, *x*-axis) and was measured for each compound treatment (330 compounds tested at 3 concentrations; 100, 300 and 1000 nM; *y*-axis) with compound hits selected (all 16 displayed) as those with >50.5% phenotypic similarity to control after 24 h treatment. (**B**) Hierarchical clustering and heatmap visualization of 50 PCs (after min–max scaling) showing separation of the *SORL1*^−/−^ NPCs after treatment with different categories of drug. (**C**) Violin plots depict phenotypic signatures following treatment of *SORL1*^−*/−*^ NPCs with the 16 hit compounds with reference to vehicle-treated *SORL1*^−*/−*^ and wild-type NPCs. Signatures are based on unscaled PC values for PCs 1–50; significant separation was observed between vehicle-treated *SORL1*^−*/−*^ NPCs and morphological signature induce by treatment with the 16 hit compounds. NPC, neural progenitor cell; PC, principal component; PCA, principal component analysis; WT, wild-type.

A three-layer neural network (NN) classification with two-fold cross-validation was used to identify compounds that induced a morphological profile similar to that of the wild-type controls in the *SORL1*^−/−^ NPCs. NN classification performance/accuracy for the screening results was assessed based on the DMSO-treated controls using a confusion matrix, in addition to measures of sensitivity, specificity and detection rate (Supplementary Fig. 4B and Supplementary Table 6). Images from the positive control class were >99.68% likely to classify as a wild-type NPCs, while those from the negative control class were >99.64% likely to classify as *SORL1*^−/−^ NPCs (Supplementary Table 7). The NN classification model was used to output a similarity score between 0.0 and 1.0 for every compound-treated sample for each of the three *SORL1*^−/−^ NPC sub-clones at each concentration (100, 300 and 1000 nM). A score of 1.0 denotes 100% phenotypic similarity to the vehicle-treated wild-type controls with 0% similarity to the vehicle-treated *SORL1*^−/−^ NPCs (negative controls); hits were defined as those compound-treated samples with a score of >0.505 after classification. Sixteen compounds induced morphology in *SORL1^−/−^* NPCs that scored >50.5% similarity to wild-type controls (replicated in *n* = 3 sub-clones at one or more of the concentrations tested, Fig. 5A) suggesting partial rescue of the mutant phenotype by these drugs.

The 16 compounds corresponded to 14 unique drug treatments; two of the 16 hits (topotecan and ixazomib) were represented with two formulations (ixazomib/ixazomib citrate and topotecan/topotecan HCl). According to library annotation, those 16 compounds were grouped into 3 classes: metabolism, protease/proteasome inhibitors and DNA synthesis/repair inhibitors (Supplementary Table 8; Fig. 5B). Phenotypic signatures induced by compounds from the same regulatory target annotation were hierarchically clustered, suggesting an overlapping morphological profile for compounds targeting each of the regulatory processes (Fig. 5C). Phenotypic profiles similar to the wild-type controls were observed in *SORL1*^−/−^ NPCs upon treatment with the hit compounds (Fig. 5B and C) and all 16 hit compounds induced morphological profiles in knock-out lines that were significantly different to those of the negative control class (*P <* 0.05, Bray–Curtis dissimilarity test, Fig. 5C). PCs with the greatest predictive power in each of the classification models were ranked by relative importance (Supplementary Table 9). The top-ranked components, PC01, PC12 and PC07 had greatest predictive power in the neural network suggesting the most important features for the classification represented measures of plasma membrane, Golgi apparatus, mitochondrial, endoplasmic reticulum and cytoskeletal texture and radial distribution and nuclear eccentricity, shape, area, radial distribution and compactness. Plots of these PCs in 3D show phenotypic separation of controls (Supplementary Fig. 4C). These cellular components showed enrichment in the SORLA NPC-expressed protein network described above (e.g. Golgi apparatus GOCC:0005794, Hypergeometric test, ES = 0.81, FDR, *P* = 5.9 × 10^−6^) (Supplementary Table 10). Additionally, enrichment of *SORL1* interaction network was observed in cellular components linked to the image-based phenotypic features relevant to SORLA function (e.g. the most significant enrichment was in early endosome GOCC:0005769, Hypergeometric test, ES = 1.47, FDR, *P* = 7.29 × 10^−15^).

### Compound–protein interaction networks are enriched in DNA repair, metabolism and protease regulation pathways

**Figure 6:**
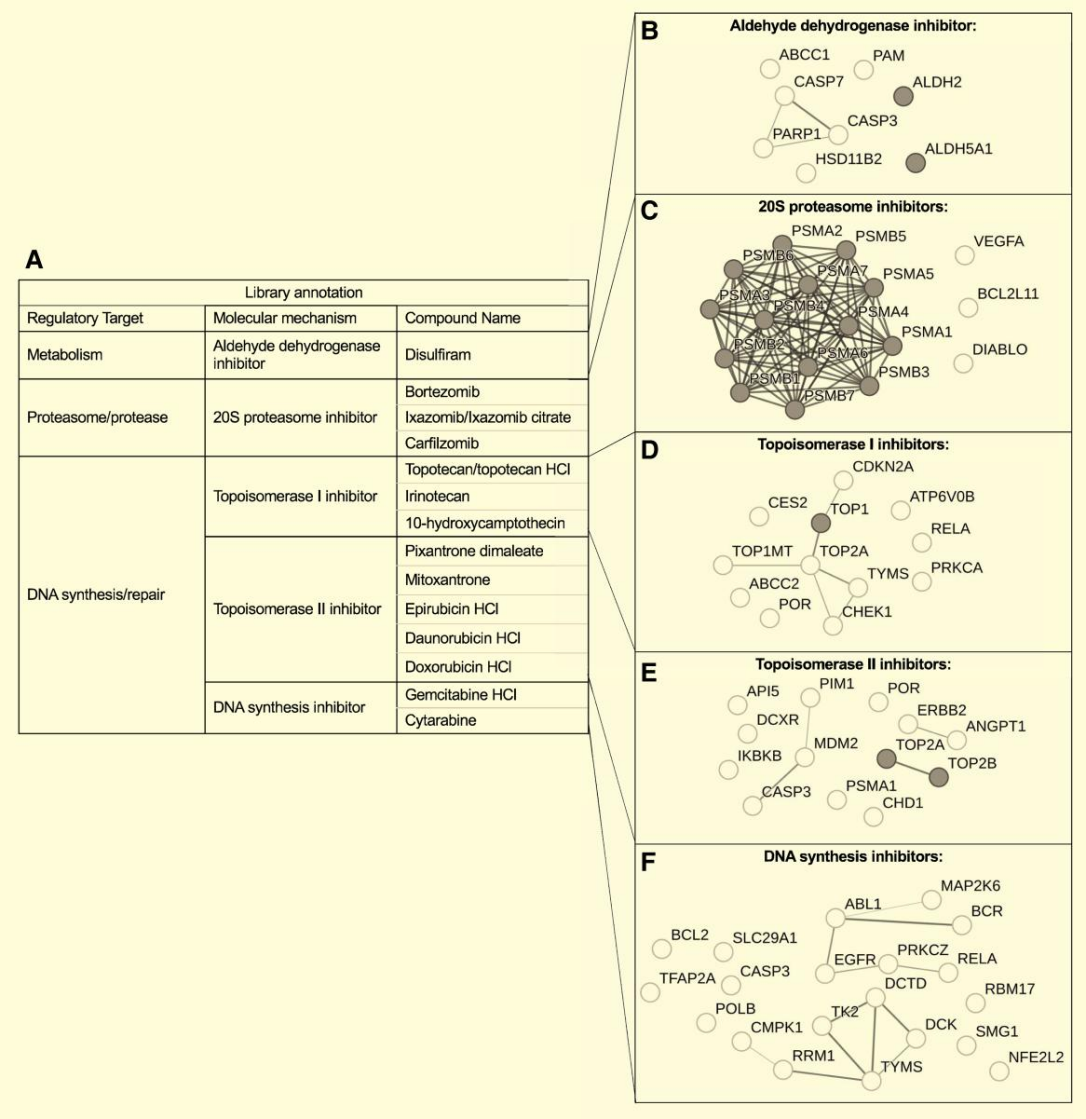
**The 16 hit compounds which rescue effects of SORLA depletion on NPC morphology**. Sixteen inhibitor compounds regulating three biological pathways/targets from five mechanistic classes from the 330-compound library were identified from the pilot drug screen. Those 16 compounds represent 14 FDA-approved drugs, since 2 formulations of topotecan and 2 of ixazomib were identified. (**A**) The table summarizes the 14 drugs according to regulatory target and annotated molecular mechanism. (**B–F**) STRING (protein only) network displayed with grey nodes reflecting those proteins interacting directly with compounds based on STITCH database (compound–protein interaction network). Network diagram lines indicate physical interaction based on data from experimental evidence and databases. Line weight/thickness indicates confidence of interaction. FDA, Food and Drug Administration; HCl, hydrochloride; NPC, neural progenitor cell.

To understand the molecular targets and pathways associated with the phenotypic response of *SORL1^−/−^* NPCs to the hit compounds, we performed a STITCH-STRING database search for experimentally validated compound–target interactions for the 14 distinct drug molecule hits grouped by regulatory class (Fig. 6A). This confirmed that disulfiram targets aldehyde dehydrogenases (Fig. 6B) bortezomib, carfilzomib and ixazomib target proteasome-related genes (Fig. 6C), and the remaining compounds target topoisomerase I (Fig. 6D), topoisomerase II (Fig. 6E) which inhibit TOP1 and TOP2A/2B respectively. Compounds inhibiting DNA synthesis (gemcitabine HCl and cytarabine) targeted the products of multiple genes expressed in our NPCs (Fig. 6F). There were common targets for the compounds within each mechanistic class (Supplementary Fig. 5). This data agrees with the functional/mechanistic annotation for the list of predicted hits provided with the compound library.

Enrichment analysis was performed on the protein-protein interaction network associated with each group of compounds within the STRING database. The top eight targets expressed in NPCs for the aldehyde dehydrogenase inhibitor disulfiram included two genes from the family encoding aldehyde dehydrogenases (*ALDH2*, *ALDH5A1*). The most significant GO biological process and molecular function terms for the disulfiram network were response to corticosteroid (GO:0031960, Hypergeometric test, ES = 1.77, FDR, *P* = 0.0058) and cysteine-type endopeptidase activity involved in the execution phase of apoptosis (GO:0097200, Hypergeometric test, ES = 2.74, FDR, *P =* 0.0195), respectively (Fig. 6B).

The proteasome inhibitors bortezomib, carfilzomib and Ixazomib target a network of 18 proteins expressed in our NPCs, including 15 genes encoding sub-units of the 20S proteasome (*PSMA1-7* and *PSMB1-8*). The most significant GO biological process and molecular function terms for the bortezomib, carfilzomib and ixazomib network were proteasomal ubiquitin-independent protein catabolic process (GO:0010499, Hypergeometric test, ES = 1.64, FDR, *P* = 8.31 × 10^−18^) and threonine-type endopeptidase activity (GO:0004298, Hypergeometric test, ES = 2.70, FDR, *P* 7.39 × 10^−39^), respectively (Fig. 6C).

The compounds regulating DNA synthesis/repair pathways belong to a number of mechanistic classes. The topoisomerase I inhibitors topotecan, irinotecan and 10-hydroxycamptothecin target a network of 12 proteins expressed in our NPCs including topoisomerase I (TOP1). The most significant GO biological process and molecular function terms for this network were DNA topological change (GO:0006265, Hypergeometric test, ES = 2.69, FDR, *P* = 0.00077) and DNA topoisomerase activity (GO:0003916, Hypergeometric test, ES = 2.85, FDR, *P* = 0.00010) respectively (Fig. 6D). The topoisomerase II inhibitors epirubicin, doxorubicin, daunorubicin, pixantrone and mitoxantrone target a network of 15 proteins expressed in our NPCs including topoisomerase II A and B (TOP2A and TOP2B). The most significant GO biological process and molecular function terms for this network were negative regulation of apoptotic process (GO:0043066, Hypergeometric test, ES = 0.71, FDR, *P* = 0.00888) and DNA topoisomerase type II (GO:0003918, Hypergeometric test, ES = 2.94, FDR, *P* = 0.0266), respectively (Fig. 6E). Cytarabine and gemcitabine were associated with network of 20 proteins including multiple proteins associated with DNA biosynthetic processes (TK2, DCTD, TYMS and DCK) expressed in our NPCs (Fig. 6F). However, the annotated mechanism of action for these compounds is not via protein–compound interaction but rather intercalation/incorporation into DNA during replication in S-phase of the cell cycle. In keeping with this, the network associated with cytarabine and gemcitabine was most enriched in deoxyribonucleoside monophosphate biosynthetic process (GO:0009157, Hypergeometric test, ES = 2.64, FDR, *P* = 8.14 × 10^−6^) and transferase activity (transferring phosphorus-containing groups, GO:0016772, Hypergeometric test, ES = 1.02, FDR, *P* = 3.4 × 10^−5^). Overall, this analysis showed that the NPC- expressed protein targets for each drug largely fell into the expected gene families, mechanistic pathways and GO processes/functions, given the drug functions.

### Structural similarity and computational ligand-based prediction of novel biological targets and mechanistic pathways

**Figure 7:**
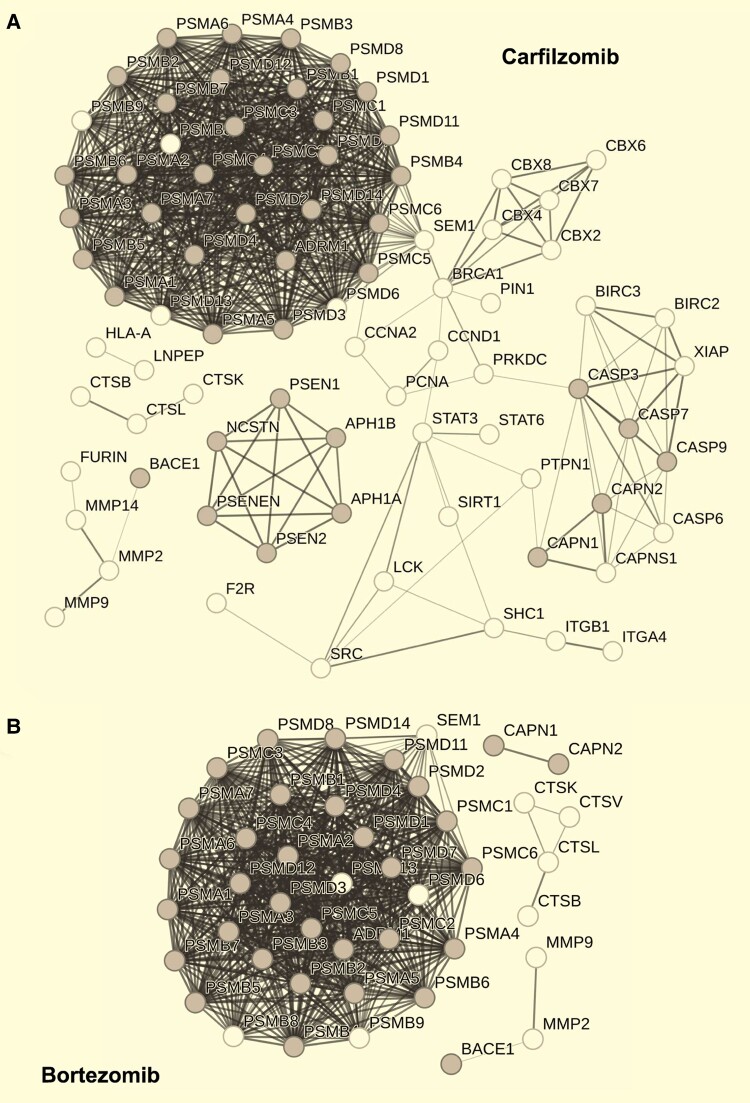
**Predicted compound–protein interaction networks based on structural similarity are enriched in Alzheimer’s disease KEGG pathway**. The proteasome inhibitors (**A**) carfilzomib and (**B**) bortezomib were predicted to target multiple sub-units of the 20S proteasome based on structural similarity. Enrichment analysis showed multiple nodes in the target network for each compound was enriched in the KEGG pathway for Alzheimer’s disease (hsa05010). Nodes in grey depict those molecular targets which are both part of the predicted compound target and feature in the gene list for KEGG pathway hsa05010. Lines indicate physical interaction based on data from experimental evidence and databases. Line weight/thickness indicates confidence of interaction. KEGG, Kyoto Encyclopedia of Genes and Genomes.

Since compounds may have previously unreported off-target effects we also explored mechanism of action using structural similarity ligand-based target prediction. We used the Similarity Ensemble Approach (SEA) search tool to query compound–target databases with the SMILES notation of the chemical structure of our 14 drug hits. Network and enrichment analysis were then applied to expand the compound–target search space to derive a set of novel computationally-predicted compound–protein interactions for each drug. There were sufficient interactions predicted for NPC-expressed genes to predict protein-protein interaction networks for carfilzomib, bortezomib, disulfiram, mitoxantrone, irinotecan, cytarabine and daunorubicin/doxorubicin/epirubicin (which are molecular analogues) (Supplementary Table 11). Target networks for proteasome inhibitors carfilzomib (Fig. 7A) and bortezomib (Fig. 7B) were significantly associated with the KEGG pathway for Alzheimer’s disease hsa05010; carfilzomib: Hypergeometric test, ES = 1.26, FDR, *P* = 5.13 × 10^−38^ and bortezomib: Hypergeometric test, ES = 1.56, FDR, *P* = 7.54 × 10^−44^. These networks included *BACE1* and *CAPN1/2* within the networks for both carfilzomib and bortezomib, as well as *PSEN1/2* in the network for bortezomib only. These findings together suggest network nodes likely to be disrupted in *SORL1*^−/−^ NPCs are associated with SORLA- and Alzheimer’s disease- relevant biological processes, cellular compartments and pathways. One gene, *BACE1,* was expressed in our NPCs and part of the *SORL1* network of physical interactors. *BACE1* is also associated with Alzheimer’s disease via the literature and in the GO, KEGG and DISEASES database and in this study we show it is a predicted target of bortezomib and carfilzomib (based on SEA search). The null hypothesis that any single gene could be common to all of the above due to chance could not however be rejected [Hypergeometric probability, *P*(*X* = 1) = 0.05971].

## Discussion

Drug development for dementia has had an extremely high rate of attrition over many decades.^38,39^ While recent progress, with the immunotherapy drugs appears promising,^8,10,40^ efficacy and minimizing off-target and adverse reactions requires further development^9,41^ and there is a need for alternative therapeutic approaches. One possible reason for the previous high failure rate is development in animal models, which do not faithfully recapitulate human disease. The use of neural derivatives of human iPSCs may increase the likelihood of success, as compounds are tested on human cells during discovery and development, facilitating early efficacy testing in the context of human physiology, and early recognition of human-specific toxicity. We used loss of SORLA in an NPC-based drug screening model because of strong genetic and functional evidence supporting *SORL1*/SORLA’s involvement in Alzheimer’s disease. The previous finding of image-based endosomal phenotypes in *SORL1^−/−^* NPCs provided the rationale for the cell type. The use of a line from a healthy individual facilitated a screen for compounds that cause reversion of the phenotype caused by the loss of SORLA to that of a healthy cell. Finally, we used Cell Painting because this high-content analysis captures the rich phenotypic information present in image data. Thus, compounds that result in reversion of multiple phenotypic features induced by loss of SORLA loss are identified without these phenotypes having to be previously defined. Such methodology has been successfully applied to cancer drug screening.^34,42,43^

Here, we demonstrate that Cell Painting distinguishes wild-type hiPSC-derived NPCs from those lacking SORLA. The principal component with the greatest predictive power in these classification models represented ER, Golgi apparatus and plasma membrane measures, consistent with SORLA’s role in intracellular trafficking. These reference profiles permitted identification of 16 compounds from a library of 330 FDA-approved drugs where there was evidence for reversion of the mutant phenotype at one or more of the concentrations tested. These compounds induced changes in multiple cellular/sub-cellular compartments, including the nucleus, mitochondria, endoplasmic reticulum, Golgi apparatus and cell membrane, suggesting the high-content approach identifies compounds with diverse target proteins/pathways and mechanism of action. The hits included four inhibitors of proteasome/proteases, eight topoisomerase inhibitors, three DNA synthesis inhibitors and an aldehyde dehydrogenase inhibitor, representing three biological pathways: DNA damage/repair, metabolism and protein degradation.

Aldehyde dehydrogenase 2 (ALDH2) activity is important for aldehyde metabolism, e.g. it catalyses the rate-limiting step of alcohol metabolism, and has been linked to multiple neurological and neurodegenerative diseases.^44^ In mice, ALDH2 regulates neuroinflammation and Aβ-levels *in vivo*, particularly in response to ethanol.^45^ Modification of this pathway, e.g. using chemical compounds related to disulfiram may, therefore, be useful in the regulation of this aspect of Alzheimer’s neuropathology.^44^

SORLA has not been linked to DNA damage/repair, but depletion of family member SORCS2 led to increased DNA double strand breaks in the mouse dentate gyrus and higher numbers of topoisomerase IIβ-dependent breaks in human dopaminergic neurons.^46^ There is also mounting evidence for a relationship between DNA damage and neurodegeneration.^47,48^

As inhibition/impairment of the proteasome has been associated with Alzheimer’s disease,^49,50^ it was unexpected that drugs inhibiting the proteasome rescued *SORL1*^−/−^ associated morphology. This may be due to a differential impact of proteasome inhibition in NPCs and neurons, or it may point to a previously uncharacterized mechanism of action of the drug(s). Our analysis revealed a number of non-proteasome linked, Alzheimer’s disease-associated predicted targets of the drug hits. For example, production of Aβ depends on proteolysis of APP by β- and γ-secretases,^51,52^ and β-secretase (which is NPC-expressed) is a predicted target of proteasome inhibitors bortezomib and carfilzomib. Incorporation of bortezomib into amyloidosis treatment significantly improved patient outcomes in clinical trials.^53,54^ Such instances demonstrate that pharmacological confirmation of ligand–target interaction is required for translation of possible lead compounds.

In terms of limitations, screening a library of small molecules enriched for anti-cancer compounds is an atypical choice for an Alzheimer’s disease study. Morphological features associated with SORLA depletion are similar in NPCs and neuronal cell types.^19,20^ Loss of Sorla in mice leads to increased proliferation of NPCs,^55^ supporting the selection of a library with many small molecules influencing cell proliferation. Given the pleiotropic effects of most drugs, as demonstrated in the SEA search presented here, exploring the morphological response of NPCs to a library with broad mechanistic classes may reveal mechanisms for such compounds beyond their annotated function in cell-cycle regulation. Comprehensive drug screening assays should include replication of compound hits from other mechanistic classes both independent from and related to the library used here. This was a pilot screen, and, as such, was performed in a single cell line and type. Future work should be performed in other neural cell types also using additional biological donor cell lines, including a female line.

As compounds that cause reversion of the mutant phenotype do not act by altering SORLA directly, the precise mechanisms by which they elicit their response remains uncharacterized. The results of our network analysis support future deconvolution of molecular targets, which is required for the necessary mechanistic understanding, and a clear understanding of off-target effects, which is required for compound safety. The *in silico* networks identified in this study suggest possible targets for future target–ligand interaction and binding affinity assays, whilst insights from the pathway/enrichment analysis will support exploration of the mechanisms responsible for the phenotypic reversion we observed.

Alzheimer’s disease pre-clinical research is largely focussed on mature neural cells, such as neurons and/or glia. This is logical given that these cells are relevant to the disease mechanisms as they are currently understood.^14^ But recent evidence suggests NPCs are also important in Alzheimer’s disease. Proliferative, pluripotent NPCs persist in the ageing brain^33^ and there is a greater decrease in adult hippocampal neurogenesis in Alzheimer’s disease cases than in healthy controls.^32^ Our findings in *SORL1*^−/−^ NPCs are in keeping with this: there were fewer nuclei positive for the multipotent neural stem cell marker Sox2 than in wild-type cultures. This suggests that SORLA loss in NPCs may lead to alterations in the levels of pluripotent NPCs (which may be relevant during development and/or adulthood).

In summary, our goal was to apply morphological profiling via Cell Painting to differentiate wild-type NPCs from those lacking SORLA, and thus develop a drug screening assay to discover compounds for future translation for Alzheimer’s disease. We discovered a morphological signature that distinguishes neural progenitors lacking *SORL1* from wild-type isogenic controls and demonstrated that this hypothesis-free assay has the potential for drug screening. A set of putative hits was identified, but follow-up studies to confirm their effect and potency and their targets/mechanisms of action, is needed to determine their translational potential. In the future, this methodology could be used to screen larger, more mechanistically diverse drug libraries for novel drug discovery in Alzheimer’s disease.

## Supplementary Material

fcae101_Supplementary_Data

## Data Availability

Raw quantitative datasets that can be used for independent verification of results are available in supplementary information. The source code and algorithm used for data processing and neural network classification is built into the HC analysis module of StratoMineR (Core Life Analytics), a proprietary licensed software.
