# Supplementary material for: Morphological profiling in human neural progenitor cells classifies hits in a pilot drug screen for Alzheimer’s disease
Source: Brain Commun. 2024 Mar 28;6(2):fcae101. doi: 10.1093/braincomms/fcae101 (PMC10994270; doi:10.1093/braincomms/fcae101)
Supplement: fcae101_Supplementary_Data [file fcae101_supplementary_data.zip › Supplementary_figures.pdf]

Supplementary Figures

Morphological profiling in human neural progenitor cells classifies hits in a pilot drug screen for Alzheimer's disease

Amina H. McDiarmid[1], Katerina O. Gospodinova[1], Richard J.R. Elliott[2], John C. Dawson[2], Rebecca E. Hughes[2], Marie-Therese El-Daher[3], Susan M. Anderson[1], Sophie C. Glen[1], Simon Glerup[4], Neil O. Carragher[2] and Kathryn L. Evans[1]

[1]Centre for Genomic & Experimental Medicine, Institute of Genetics & Cancer, University of Edinburgh, Western General Hospital, Crewe Road, Edinburgh EH4 2XU, United Kingdom

[2]Cancer Research UK Scotland Centre, Institute of Genetics & Cancer, University of Edinburgh, Western General Hospital, Crewe Road, Edinburgh EH4 2XU, United Kingdom

[3]Medical Research Council Human Genetics Unit, Institute of Genetics & Cancer, University of Edinburgh, Western General Hospital, Crewe Road, Edinburgh EH4 2XU, United Kingdom

[4]Department of Biomedicine, Aarhus University, Høegh-Guldbergs Gade 10, building 1116, 250, 8000 Aarhus, Denmark

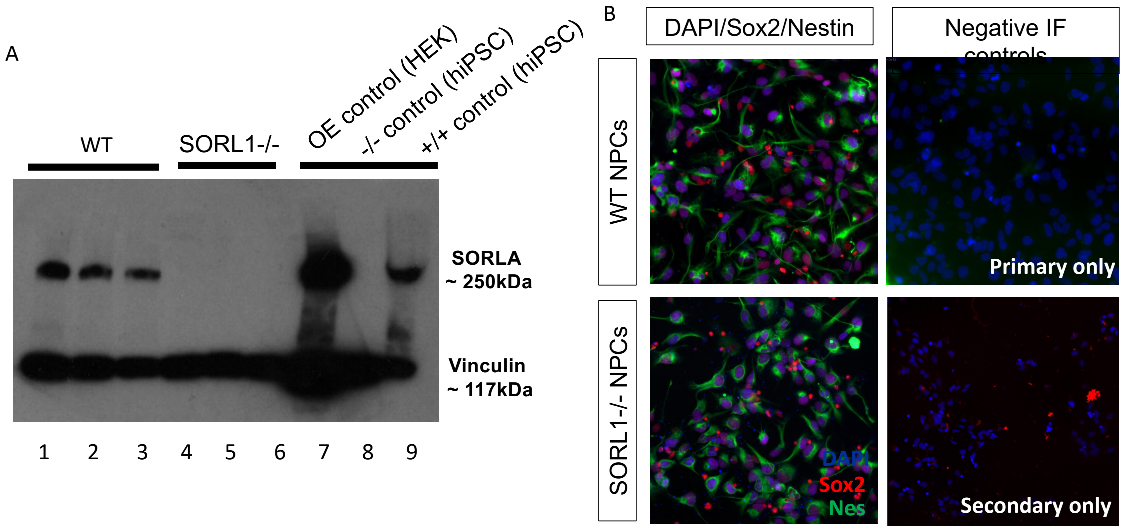

Supplementary Figure 1 | Immunoblot for SORLA in neural progenitors derived from SORL1-/- and wild-type hiPSCs.

A) Mutations were introduced in iPSCs by non-homologous end-joining using a CRISPR/Cas9 guide RNA targeted to exon 31 of the SORL1 gene. Neural induction of iPSCs to NPCs was performed using a neural induction kit with SMAD inhibition (embryoid body method, Stem Cell Technologies). This method resulted Sox+/Nestin+ NPCs in which depletion of SORLA expression beyond levels detectable by immunoblotting was observed. For this immunoblot, Vinculin was used as loading control (lane 1-3 = unedited wild-type control, lane 2 and 3 = wild-type CRISPR controls, lanes 4-6 = multiple SORLA depleted subclones, lane 7 = SORLA overexpression in HEK cells as a positive control, lane 8 = a negative control from SORL1-/- hiPSCs and lane 9 = wild-type hiPSCs as a positive control). B) Representative colour composite images of NPCs from wild-type and SORL1 -/- with immunocytochemistry used to detect Sox2 (red) and Nestin1 (green) with DAPI (blue) used as a nuclear stain. *Abbreviations: WT, wild-type; DMSO, dimethyl sulfoxide; RNA, ribonucleic acid; Con. A, Concanavalin A, WGA, Wheat Germ Agglutinin); NPC, neural progenitor cell; DAPI,* 4′,6-diamidino-2-phenylindole.

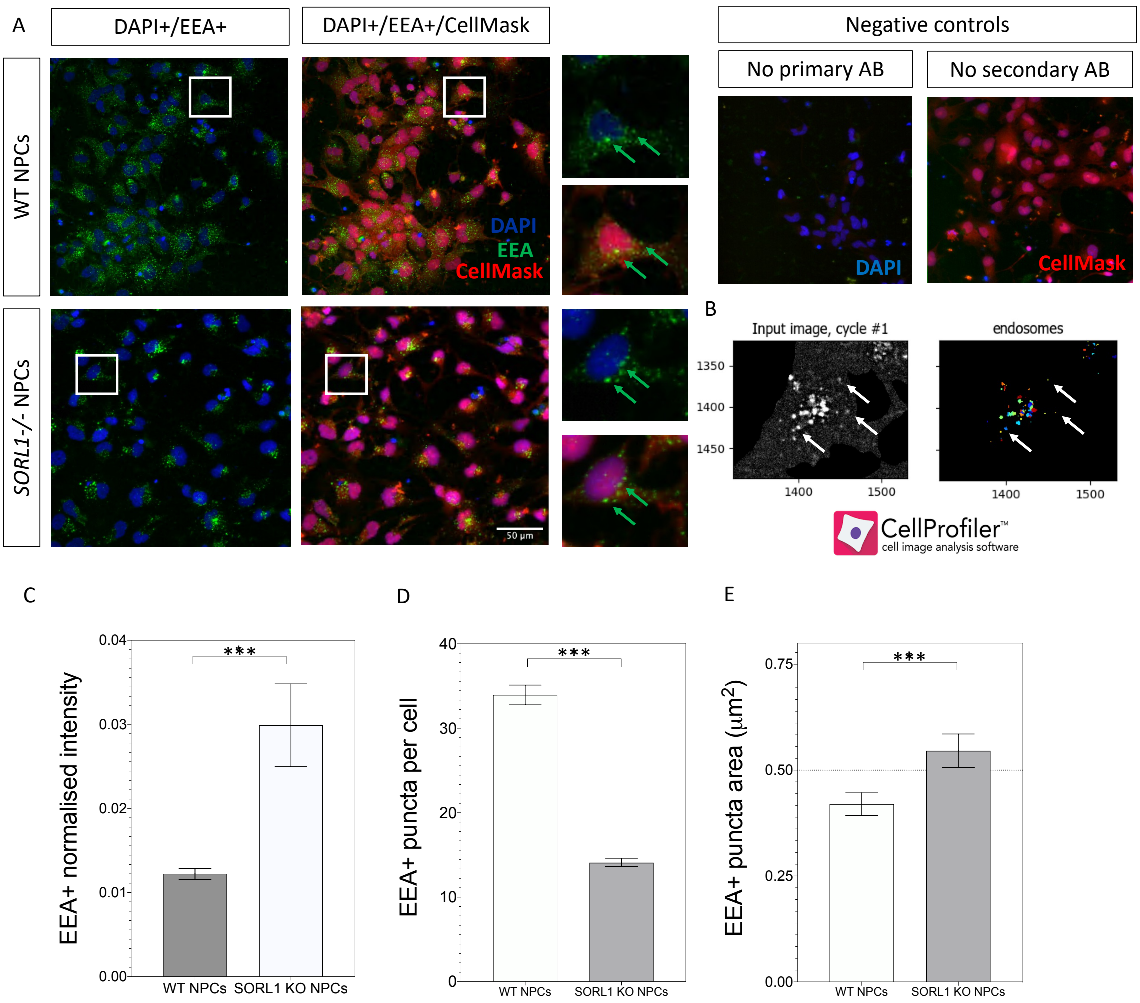

Supplementary Figure 2 | Early endosome antigen 1 (EEA1)-positive puncta are enlarged in SORL1-/- NPCs

A) Representative individual channel and composite images of NPCs from wild-type and SORL1 -/- with immunocytochemistry used to detect early endosome marker early endosome antigen 1 (EEA1) with DAPI and HCS CellMask used as a nucleus and nucleus/cytoplasmic counterstain respectively. (B) Example images from CellProfiler image analysis software demonstrating the adaptive thresholding strategy used to automate detection of EEA+ puncta from immunofluorescence microscopy images supporting quantification of puncta count, intensity and area. (C) Graph showing increase in integrated intensity of EEA+ puncta normalized to cell count (mean intensity) between WT and SORL1-/- NPCs. (D) Total EEA+ puncta count per cell was increased in wild-type NPCs compared to SORL1-/- NPCs. (E) EEA+ puncta area was increased in SORL1-/- NPCs with greater frequency of puncta >0.5um2 compared to wild-type NPCs demonstrating early endosome enlargement as a result of SORLA depletion. Scale bar = 50um. Error bars show standard error of the mean. *** p < 0.0001, Mann-Whitney U-Test to compare ranks used as data has unequal variance and did not display a normal distribution. *Abbreviations: AB, antibody; WT, wild-type; EEA1, early endosome antigen 1; NPC, neural progenitor cell; DAPI, 4',6-diamidino-2-phenylindole.*

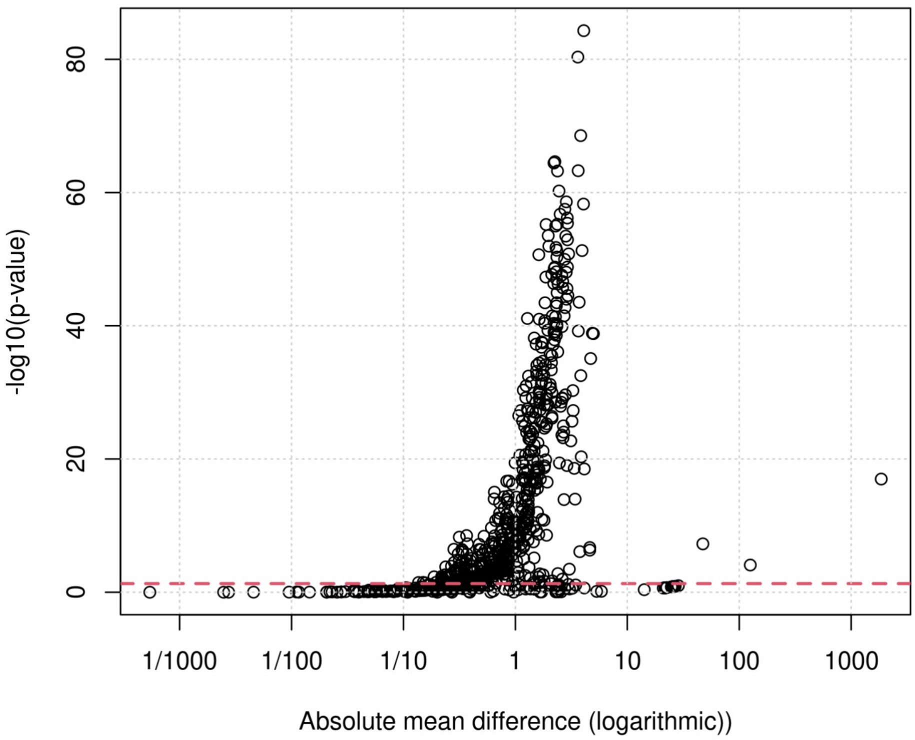

Supplementary Figure 3 | Volcano-like plot to visualise feature-level differences in wild-type versus SORL1-/- NPCs from the pilot drug screen.

Direct comparison of all 756 features used in the classification demonstrates 408 features are significantly different between vehicle-treated wild-type and SORL1-/- control NPCs (t-test with Benjamini-Hochberg control for False Discovery Rate). X-axis shows the –log10(p-value) resulting from the t-test with BH correction for FDR. Y-axis shows the absolute mean difference plotted on a logarithmic scale as a proxy for fold-change. Red dashed line denotes the significance threshold (FDR-corrected, $p = 0.05$). Each data point represents a feature measured as part of the Cell Painting assay. Features are not labelled due to large number of data points. Abbreviations: FDR, false-discovery rate; NPCs, neural progenitor cells.

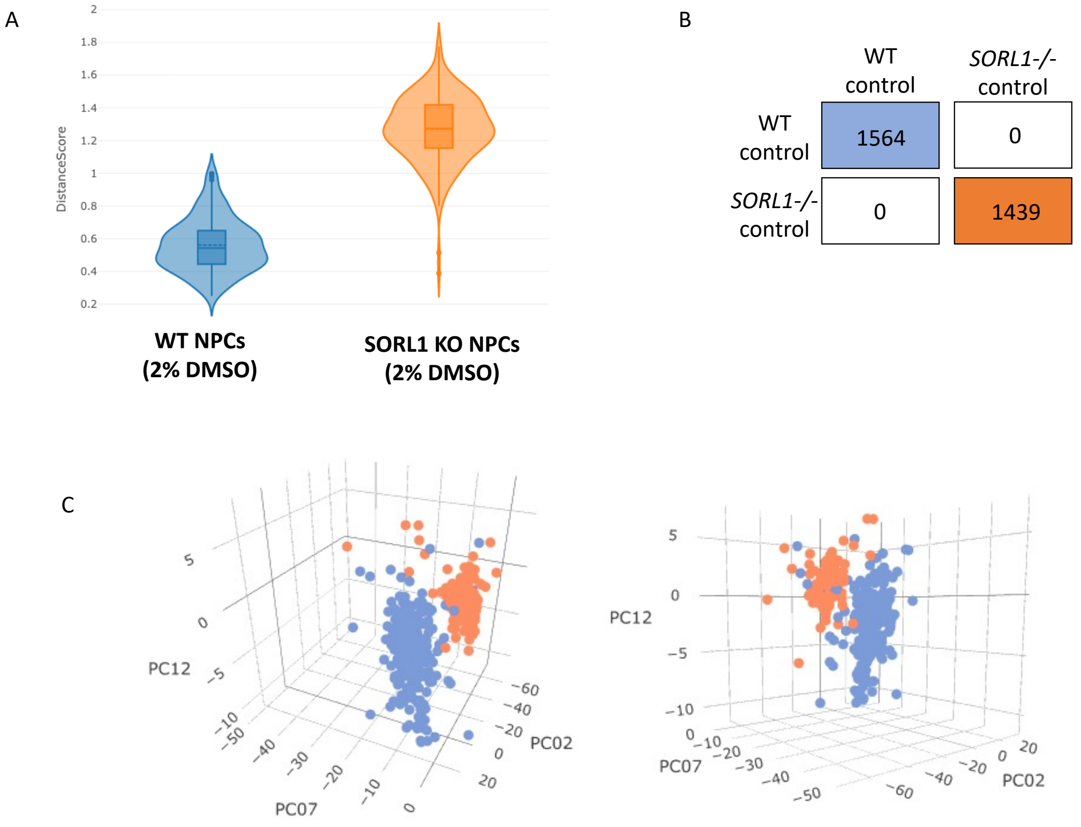

Supplementary Figure 4 | Phenotypic separation of the screening controls comparing vehicle-treated SORL1-/- NPCs to isogenic wild-type controls.

PCA was applied to 756 analytical features to reduce dimensionality and identify factors explaining variation between positive and negative classes corresponding to DMSO-treated WT and DMSO-treated SORL1-/- NPCs. The positive and negative control classes were visualized using three-dimensional scatter plots. (A) Violin plots representing the Bray-Curtis dissimilarity scores from the WT and SORL1 KO NPC classes used to assess significance using a non-Euclidean distance measure of phenotypic separation. (B) Confusion matrix demonstrating the accuracy of classification by Neural Network for actual and predicted WT and SORL1-/- control images. (C) Two orthogonal views of the same 3-dimensional scatter plot with PCA12 (x-axis), PCA07 (y-axis) and PCA02 (z-axis) demonstrate separation of the negative (orange, SORL1 KO NPCs) and positive (purple, WT NPCs) control classes based on PCs extracted from the analysis. Each data point in the 3D scatter plot represents the well-level median (aggregated from 4 image-level replicates per well) for 24 and 16 well-level replicates acquired from WT and SORL1-/- NPCs respectively. Abbreviations: WT, wild-type; DMSO, dimethyl sulfoxide; NPC, neural progenitor cell; PCA, principal component analysis; PC, principal component.

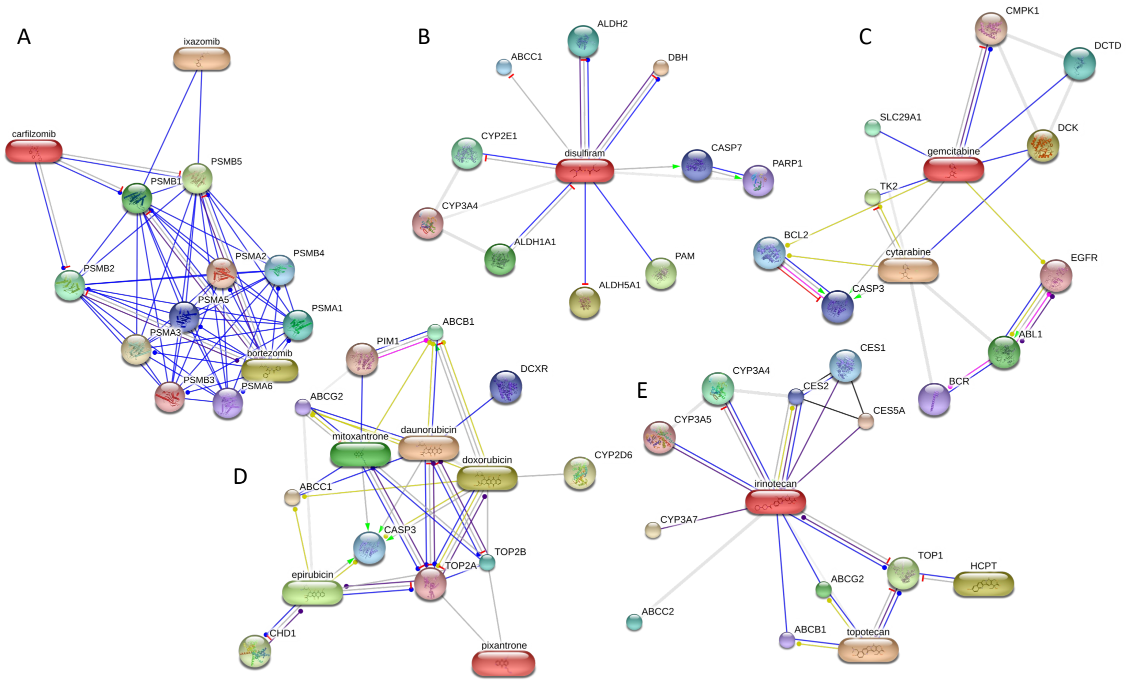

Supplementary Figure 5 | Network visualization of compound hits grouped by mechanistic class using STITCH-STRING database confirms targeting to annotated proteins.

The 14 drug hits were from 5 mechanistic classes. Network analysis using the STITCH-STRING database was used to explore experimentally validated targets per class. (A) Bortezomib and carfilzomib target the 20S proteasome. (B) Aldehyde dehydrogenase inhibitor disulfiram targeted ALDH2 and ALDH5A1. (C) DNA synthesis inhibitors gemcitabine HCl and cytarabine have no targets expressed in our NPCs since canonical action of these compound is not via protein-protein interaction. (D) Topoisomerase II inhibitors target TOP2A and TOP2B. (E) Topoisomerase I inhibitors topotecan, irinotecan and 10-hydroxycamptothecin target TOP1. All nodes were expressed in an independent line of wild-type human NPCs derived from the same parent line, QOLG-1 as determined by RNAseq analysis of transcriptome. Compound-protein interaction networks generated and visualised using STITCH-STRING database.
