# Supplementary material for: Morphological profiling in human neural progenitor cells classifies hits in a pilot drug screen for Alzheimer’s disease
Source: Brain Commun. 2024 Mar 28;6(2):fcae101. doi: 10.1093/braincomms/fcae101 (PMC10994270; doi:10.1093/braincomms/fcae101)
Supplement: fcae101_Supplementary_Data [file fcae101_supplementary_data.zip › Supplementary_materials_and_methods.pdf]

Supplementary Materials and Methods

Morphological profiling in human neural progenitor cells classifies hits in a pilot drug screen for Alzheimer's disease

Amina H. McDiarmid[1], Katerina O. Gospodinova[1], Richard J.R. Elliott[2], John C. Dawson[2], Rebecca E. Hughes[2], Marie-Therese El-Daher[3], Susan M. Anderson[1], Sophie C. Glen[1], Simon Glerup[4], Neil O. Carragher[2] and Kathryn L. Evans[1]

[1]Centre for Genomic & Experimental Medicine, Institute of Genetics & Cancer, University of Edinburgh, Western General Hospital, Crewe Road, Edinburgh EH4 2XU, United Kingdom

[2]Cancer Research UK Scotland Centre, Institute of Genetics & Cancer, University of Edinburgh, Western General Hospital, Crewe Road, Edinburgh EH4 2XU, United Kingdom

[3]Medical Research Council Human Genetics Unit, Institute of Genetics & Cancer, University of Edinburgh, Western General Hospital, Crewe Road, Edinburgh EH4 2XU, United Kingdom

[4]Department of Biomedicine, Aarhus University, Høegh-Guldbergs Gade 10, building 1116, 250, 8000 Aarhus, Denmark

Cryopreservation and iPSC culture maintenance

To revive hiPSCs from cryopreservation, cells were thawed by incubation for 2 minutes at 37°C and retrieved by centrifugation (200 x *g* for 3 minutes) prior to resuspension in Essential 8 (E8, (A1517001, Life Technologies) medium with 1x RevitaCell (A2644501, Life Technologies). Colonies were maintained on vitronectin-coated plastic culture vessels, fed daily with E8 and incubated at 37°C (95% humidity with 5% CO_2). Colonies were passaged at least twice after revival and prior to genome editing. All hiPSC cultures were confirmed as negative for mycoplasma on a monthly basis by submitting media supernatant for PCR testing by Institute of Genetics and Cancer Technical Services using a Lonza MycoAlert® kit (LT07-218).

Nucleofection and subcloning of iPSCs

Colonies of QOLG-1 wild-type parent hiPSCs were fed with E8 2 hours prior to nucleofection. For nucleofection, cells were dissociated using Accutase (A6964, Merck), counted, and $1x10^6$ cells were transfected using the Nucleofector II, Human Stem Cell kit (VPH-5012, Lonza) and the B-016 programme on the Amaxa Nucleofector II B device (Amaxa Biosystems). Pre-warmed E8 media (A1517001, Life Technologies) with 1 x RevitaCell was added following nucleofection. The cells were then incubated at 37 °C for 5 min and gently added to a vitronectin-coated 6-well plate containing 2 ml of E8 containing 1 x RevitaCell. 2 µg of the Cas9 plasmid containing the gRNA were used in each transfection. Forty-eight hours following transfection, colonies were dissociated with Accutase and retrieved from suspension by centrifugation (200g for 3 minutes). The cell pellet was resuspended in 700 µL Essential 8 Flex (E8 Flex, A2858501, Life Technologies) plus 10% CloneR™ supplement (05888, Stemcell Technologies). Single GFP-positive cells were sorted into individual wells of vitronectin-coated 96-well plates using a FACSJazz cell sorter (BD Biosciences). The single cells were allowed to expand into single-cell derived colonies by culturing for 48 hours (incubated at 37°C in 95% humidity with 5% CO_2) in 100 µL E8 Flex plus 10% CloneR. A full medium change was then performed with E8 Flex plus 10% CloneR. After 24 hours, plates were topped up with 25 µL E 8 Flex plus 10% CloneR. After 24 hours a full medium change with EI 8 Flex was repeated. Plates were then fed every 72 hours and monitored daily for the appearance of healthy colonies. After 10-14 days, colonies were passaged using 0.5 mM EDTA into a new 96-well. Once confluent, each clonal colony (subclone) was passaged again into two 96-wells to generate duplicate plates where one plate was used to generate cellular material for genomic DNA extraction. The other plate was used to maintain/expand the respective subclones for selection based on the genotyping result.

CRISPR guide and primer sequences
Primer sequences targeting *SORL1* exon 31 in iPSCs were:
CACCGTCGGTACCCGTCGCACACCC (top)
AAACGGGTGTGCGACGGGTACCGAC (bottom).

PCR and sequencing of gDNA for genotyping CRISPR products

Sequence analysis was used to confirm mutation of the *SORL1*ex31 target locus in *SORL1*$^{-/-}$ subclones (n=3) and select wild-type CRISPR (WTcr) controls (n=2) which had a normal

sequence at the target locus but were selected from subclones derived from CRISPR-Cas9 gene-editing. Primers flanked the target site at *SORL1* exon 31: forward 5’ CTGCTCAGAGCTGTGCCAGT 3’ and reverse 5’ AGCCTTCCCTGGAGGTACAC 3’.
A thermal cycler was used under the following programme: initial denaturation at 95°C for 1 min then 10 cycles of 95°C for 20 secs at 60°C- 67°C (decreased by 1°C each cycle for 30 secs - 1min at 72°C for 1min with a further 25-30 cycles of 95°C for 20 secs at 50°C- 57°C (decreased by 1°C each cycle) for 30 secs - 1min at 72°C for 1 min and a final extension at 72°C for 10 mins. Temperature was dependent on optimal annealing temperature of primers.

For Sanger sequencing the PCR product was cleaned (to remove excess primers and nucleotides) using ExoSAP-IT™ in the following reaction: 1 µL PCR Product, 1 µL ExoSAP-IT™ and 3 µL dH_2O (60 mins at 37°C followed by 20 mins at 80°C. Sequencing was performed on the ExoSAP-IT™ treated product using the following reaction mix: 5 µL of ExoSAP-IT™ treated product, 1 µL of Big Dye v3.1, 1 µL of Big Dye sequencing buffer, 1 µL Primer (stock 3.2 µM) and 2µL dH_2O using the following program: Initial denaturation at 96°C for 1 mins and then 30 cycles of 10 secs at 96°C at 5 secs at 50°C at 4 mins at 60°C. The purified products were precipitated with EDTA and ethanol for analysis on a 3130 or 3730 Genetic Analyser (Applied Biosystems).

Sequencing of predicted off-target cleavage sites

To explore off-target effects of CRISPR-Cas9 editing in subclones with confirmed homozygous mutation, Sanger sequencing was used as described above. Possible off-target CRISPR-Cas9 cleavage sites for the gRNA targeted to the *SORL1*ex31 locus were predicted computationally using CRISPR off-finder (http://www.rgenome.net/cas-offinder/) and first 10 with the highest match for both PAM (<1 mismatch) and sequence (<3 mismatches) were selected for analysis. The SORL1$^{-/-}$ subclonal lines derived from the CRISPR-Cas9 editing (n=3) were confirmed to carry no changes to loci other than the target-site at *SORL1* exon 31. In the crWT (n=2) subclones and the parent WT (n=1), no changes to the sequence were observed in any target or off-target sites tested. Primers sequences flanking the off-target sites were: S1EX31Tch2 forwards 5’ TATGGGCTTCAAAGGGGAGG 3’ and reverse 5’ TTATGCTGCATCTCCCCAGG 3’, S1EX31Tch11 forwards 5’ GAGAAGAGTGCTGGGACTGT 3’ and reverse 5’ TGGATCCCTACTGTATGGCC 3’, S1EX31Tch1 forwards 5’ GAGAGAAAATGCAGCCAGGC 3’ and reverse 5’ TGTGTTTCTCCCTTCCCCAC 3’, S1EX31Tch22 forwards 5’ CACAAAATGCCCACCCACAG 3’ and reverse 5’ TAGTAGAGAGGGGCTTTCGC 3’, S1EX31Tch11_2 forwards 5’ GGGTCTGGTGCCTGGAAG 3’ and reverse 5’ CAAGATTGCGCCACTGTACT 3’, S1EX31Tch19 forwards 5’ GAGTCCCAGAGCCACGATC 3’ and reverse 5’ TGCAGCATTAACAGAGCAGG 3’, S1EX31Tch10 forwards 5’ AATTGCCTACCTCCTCCACC 3’ and reverse 5’ GCCACGTTCTTCTGTCTGTC 3’, S1EX31Tch11_3 forwards 5’ GCAGAGTGGTGACGGACA 3’ and reverse 5’ CCCCTGAGAATGGAGGACC 3’, S1EX31Tch1_2 forwards 5’ TCTCAGCCCGGATAAGTAGG 3’ and reverse 5’ GTGTGAGTGGCCGAGAGTT 3’.

Karyotyping

After CRISPR-Cas9 editing, each *SORL1*ex31 (n=3) knock-out subclone, crWT (n=2) and the QOLG-1 wild-type parent line (n=1) were karyotyped using a commercial service that

provides information on chromosomal changes >1Mb resolution (KaryoStat Assay, ThermoFisher). Briefly, confluent hiPSCs colonies were detached from one well of a 6-well vitronectin-coated culture vessel using 0.5uM EDTA and retrieved by centrifugation (200g for 3 mins) to generate a cell pellet for karyotypic analysis.

NPC derivation, cryopreservation and maintenance

Briefly, an aggrewell-800 plate (34811,Stem Cell Technologies) was prepared by washing with anti-adherence solution (07010,Stem Cell Technologies). The aggrewell-800 plate is a 24-well culture plate each well contains 300 microwells used for the creation of EBs from hiPSCs. $3x10^6$ cells from each hiPSC line (n=3 *SORL1*ex31 knock-out subclones, n=2 crWT and n=1 parent wild-type), were resuspended in 1mL IM and added to individual wells of the Aggrewell-800 plate. The plate was then subjected to centrifugation (100g for 3 mins) to deposit the cells in the microwells (~10,000 cells/microwells, $3x10^6$ total cells per well) and fed daily with IM (half media change) for 5 days. EBs were replated on matrigel-coated 6-well plates, and fed daily (full media change) with IM and monitored for emergence of neural rosettes. After 7 days, EBs were treated for 1 hour with neural rosette selection reagent (05832,Stem Cell Technologies) prior to collection and replating into matrigel-coated 12-well plastic culture plates to establish passage 0 (p0) NPCs. On day 14, NPCs were passaged for the first time (p1) into Neural Progenitor Media (NPM) and allowed to expand to 100% confluence. At this point, NPCs were expanded to p2 and p3 prior to banking and cryopreservation in liquid nitrogen in vials of $2x10^6$ cells in NPM with 10% (v/v) DMSO.

Immunoblotting

Cells were lysed in ice cold 1% Triton lysis buffer [20 mM Tris–HCl pH 8.0, 10 mM EDTA, 1% Triton X-100 and 1× protease inhibitor cocktail (5892970001, Roche)] and protein concentration was measured using Bio-Rad BSA protein assay (5000116, Bio-Rad). Protein lysates were loaded on NuPAGE Tris–acetate 3–8% precast gels (EA03752BOX, Life Technologies) and subject to electrophoresis at 150 V for 1.5 h. Gels were transferred onto PVDF membranes at 30 V for 1.5 h. Membranes were blocked in 5% milk in 0.2% Tween-20 in TBS for 1 h at room temperature and probed with primary antibodies against SORLA (1:4000; 611680, BD Transduction Labs) and GAPDH (1:10,000; MAB374, Merck) diluted in blocking solution overnight at 4 °C. After washes (3 × 10 min) in 0.2% Tween-20 in TBS, membranes were incubated with secondary HRP-conjugated antibodies diluted 1:10,000 in blocking solution for 1 h at room temperature. After another three washes with TBS-0.2% Tween-20, blots were visualised using the Pierce ECL Plus Western Blotting Substrate (11527271, Thermo Scientific) and exposed using autoradiography film. Protein lysate obtained from HEK293 cells transfected with a plasmid overexpressing a human *SORL1* cDNA was used as a positive control.

Immunocytochemistry

NPCs were washed twice in 1xPBS after fixation, before incubation in blocking solution (50µL per well) for 1h at room temperature for permeabilization and blocking to prevent non-specific binding. Primary antibody solutions were prepared by diluting primary antibodies (at optimised concentrations for each antibody) and then applied to cells (10µL per well,

incubation at 4°C overnight). NPCs were washed twice in 1xPBS before permeabilization and blocking. Then, fluorescent-tagged secondary antibodies were diluted to 1:500 in blocking solution. After three washes in 1xPBS, the DNA counterstain DAPI (50µL per well: 300µM) was applied for 10 minutes and then NPCs were washed three further times in 1xPBS before finally adding 1xPBS (50µL per well) and a foil plate seal applied prior to imaging using florescence detection microscopy. HCS CellMask Orange (H32713, Invitrogen) was used a cellular counterstain (nucleus and cytoplasm) to identify cell area during EEA1 immunocytochemistry experiments. All steps carried out at room temperature unless stated otherwise.

Cell Profiler Image-analysis

To quantify the efficiency of neural induction of hiPSCs to NPCs, the proportion of cells expressing or co-expressing Sox2/Nestin as NPC marker(s) was quantified by CellProfiler (v4.1.2) using a pipeline applied to images acquired from immunocytochemistry. The analysis pipeline targeted the region of interest for each cell by segmenting DAPI counterstained nuclei and creating a nuclear mask which designated nuclear objects within images by optimising fluorescence intensity threshold parameters. Since Sox2 is a nuclear stain, using the nuclear mask, a secondary object was defined based on Sox2 signal (again according by optimising fluorescence intensity threshold parameters based on the FITC filter in which SOX2 was detected) within the nuclear mask region. The proportion of cells expressing DAPI or co-expressing DAPI with Sox3 counted. To isolate the region of interest for Nestin signal, the nuclear mask was used a reference point to identify cellular objects and a mask used to define area occupied by cytoplasmic Nestin stain. From this, the percentage change in Sox2/Nestin marker expression relative to the experimental controls was calculated to determine comparative changes in cell populations on different genetic backgrounds/experimental conditions. For the Cell Painting assay, the region of interest, i.e. a cell, was targeted by segmenting nuclei from images acquired in the DAPI channel to generate a nuclear mask. Using the nuclear mask as a reference point, segmentation of the cell body using the plasma membrane and cytoskeleton stain (TxRed channel) was used to create a mask for the cytoplasm. Exposure time, imaging plane and reference levels were calibrated within each experiment and kept constant with minor adjustments to the threshold set to eliminate background signal (adjustments made with respect to negative (primary/secondary antibody-free and dye-free) controls.

Transcriptomic Profiling

NPCs using the StemDiff Neural Induction Kit (IM, 08581, Stem Cell Technologies) following the embryoid-body (EB) protocol as per manufacturer's instructions as described above. Integrity of total RNA was analysed by Fragment Analyser Automated Capillary Electrophoresis (Agilent Technologies Inc, 5300) using the Standard Sensitivity RNA Analysis kit (DNF-471-0500) with DNA contamination assessed by Qubit dsDNA HS Assay kit (Q32854). RNA concentration was quantified by Qubit RNA HS Assay kit (Q32855) with 2.0 Fluorometer (Thermo Fisher Scientific Inc, Q32866). Sequencing libraries were generated from 50-100ng total RNA starting material using QuantSeq 3'mRNA-Seq Library Prep kit (FWD) for Illumina (Lexogen Inc, #015). After first and second strand synthesis, cDNA libraries were amplified for 18 cycles with adaptor and index sequences incorporated for parallel sequencing and bead-purification used to remove contaminating primers or

adapter-dimers. Libraries were quality controlled using the Agilent Bioanalyser with the DNA High Sensitivity kit, and cDNA concentrations quantified by Qubit dsDNA High Sensitivity assay. Single read (1x75bp) sequencing was performed on the NextSeq 550 platform (Illumina Inc, SY-415-1002) using the NextSeq 500/550 High-Output v2.5 kit (20024906). Raw data was converted to FASTQ format using Bcl2fastq2 (Illumina, v2.17.1.14). QuantSeq was used to map and quantify mRNA expression level using the Bluebee genomics analysis platform (www.bluebee.com/quantseq).

HPC and data handling

Analysis was performed in 100-image chunks, and the resulting datasets concatenated using Python. The quantitative multivariate object-resolution datasets derived from image analysis (by CellProfiler) were exported as .csv files and stored on University of Edinburgh datastore prior to upload to the HC analysis module included with StratoMineR (Core Life Analytics).

Network analysis

For the STITCH/STRING database analyses, the top 10 physical interactors were used for visualisation of the networks targeted by each class of compounds. For enrichment analysis, the top 20 interactors (physical subnetwork only) were exported to the STRING database (STRING; https://string.embl.de). The networks were then filtered to include only nodes (proteins) and validated edges (interactions between proteins) with experimental and database evidence to validate them. Network lists were also filtered to eliminate any proteins that did not show expression at transcript level in QOLG-1 wild-type NPCs. For the SEA search, predicted molecular targets were removed if they did not relate to a human protein, and those with a Z-score <1.96, MaxTC $\geq$0.4 and significance $p \leq 10^{-15}$ selected for further analysis. Protein-protein interaction (PPI) enrichment analysis was used to determine whether proteins in each network was greater than expected due to chance ($p < 0.05$).

Image Quality control

During data pre-processing, metadata was defined within the dataset and quality control carried to remove images with poor focus, illumination artefacts and debris. For image quality control, image quality metrics were analysed using MeasureImageQuality.py (CellProfiler, v4.2.1). Object size and shape measures can also be used as indicators of object segmentation performance and in-well debris/other imaging artifacts. Poor quality images were removed due to sparsity/high confluency, clumped cells leading to mis-segmentation during image-analysis, poor focus, in-well debris or illumination artefacts which could not be corrected for since these issues impact the precision of object-level quantification and the aggregation of the datasets to image-level medians. Positive and negative control image sets were visually inspected since these image sets form the test and training classes used for classification by machine learning.

Data normalisation and processing

Redundant variables with Pearson correlation co-efficient >0.99 ($p < 0.05$) were removed. Variables (known as features) were selected, transformed to ensure normality of distribution

across all features and normalised according to the sample median to control for inter-plate variation. Feature scaling was achieved by calculating robust Z-scores on a per-plate basis.
